# Acaricidal Mechanism of Scopoletin Against *Tetranychus cinnabarinus*

**DOI:** 10.3389/fphys.2019.00164

**Published:** 2019-03-06

**Authors:** Hong Zhou, Yong-qiang Zhang, Ting Lai, Xue-jiao Liu, Fu-you Guo, Tao Guo, Wei Ding

**Affiliations:** Institute of Pesticide Science, College of Plant Protection, Southwest University, Chongqing, China

**Keywords:** *Tetranychus cinnabarinus*, scopoletin, GPCR, BAG, GUK, Ca^2+^ homeostasis, calcium signaling pathway

## Abstract

Scopoletin is a promising acaricidal botanical natural compound against *Tetranychus cinnabarinus*, and its acaricidal mechanism maybe involve calcium overload according to our previous study. To seek potential candidate target genes of calcium overload induced by scopoletin in *T. cinnabarinus*, RNA-seq was utilized to detect changes in transcription levels. 24 and 48 h after treatment, 70 and 102 differentially expressed genes were obtained, respectively. Target genes included 3 signal transduction genes, 4 cell apoptosis genes, 4 energy metabolism genes, and 2 transcription factor genes. The role of 3 calcium signaling pathway-related genes, namely, G-protein-coupled neuropeptide receptor, Bcl-2 protein and guanylate kinase (designated *TcGPCR*, *TcBAG*, and *TcGUK*, respectively) in the calcium overload were investigated in this study. RT-qPCR detection showed that scopoletin treatment upregulated the expression level of *TcGPCR* and downregulated the expression level of *TcBAG* and *TcGUK*. The result of RNAi indicated that downregulation of *TcGPCR* decreased susceptibility to scopoletin, and downregulation of *TcBAG* and *TcGUK* enhanced susceptibility to scopoletin. Functional expression in Chinese hamster ovary cells showed that scopoletin induced a significant increase in intracellular free calcium [Ca^2+^]i levels by activating *TcGPCR.* These results demonstrated that the acaricidal mechanism of scopoletin was via disrupting intracellular Ca^2+^ homeostasis and calcium signaling pathway mediated by GPCR, BAG, and GUK.

## Introduction

The carmine spider mite, *Tetranychus cinnabarinus*, is one of the most polyphagous arthropod herbivores and feeds on more than 100 plant species, such as food and economic crops, ornamental plants, and weeds (Zhang et al., 2004; Çakmak et al., 2005; Sarwar, 2013). The carmine spider mite is parthenogenic and exhibits strong adaptability and fecundity. Moreover, this type of mite is one of the most difficult pests to control because it easily develops resistance to pesticides (Cruz et al., 2013).

Scopoletin (Supplementary Figure S1) is a kind of botanical natural phenolic coumarin (Supplementary Figure S2) and an important member of the group of phytoalexins isolated from many plants, such as *Erycibe obtusifolia* (Pan et al., 2011), *Aster tataricus* (Ng et al., 2003), *Foeniculum vulgare* (Kwon et al., 2002), *Artemisia annua* (Tzeng et al., 2007), *Sinomonium acutum* (Shaw et al., 2003), and *Melia azedarach* (Carpinella et al., 2005). Studies have shown that scopoletin has a wide spectrum of biological activities, such as acaricidal (Zhou et al., 2017), anti-inflammatory (Ding et al., 2009; Jamuna et al., 2015), antitumoral (Cassady et al., 1979), antioxidative (Shaw et al., 2003), hepatoprotective (Cassady et al., 1979), insecticidal (Tripathi et al., 2011), antifungal (Prats et al., 2006), and alleopathic properties (Pérez and Nuñez, 1991). Especially, a previous study found that scopoletin exhibits excellent contact killing, as well as systemic, repellent, and oviposition inhibition activities against *T. cinnabarinus* (Zhou et al., 2017). Moreover, mites did not develop resistance against scopoletin after 18 generations possibly because of the multi-target mechanism of scopoletin against *T. cinnabarinus* (Zhang et al., 2011). Furthermore, after exposure to scopoletin, several typical neurotoxic symptoms, such as excitement and convulsions, were observed in mites, and the compound specifically inhibits the nervous system targets, AChE, Na^+^-K^+^-ATPase, Ca^2+^-Mg^2+^-ATPase, and Ca^2+^-ATPase, which indicates that scopoletin is a neurotoxin, in which Ca^2+^ plays a key role as an intracellular second messenger (Liang et al., 2011; Hou et al., 2015).

**FIGURE 1:**
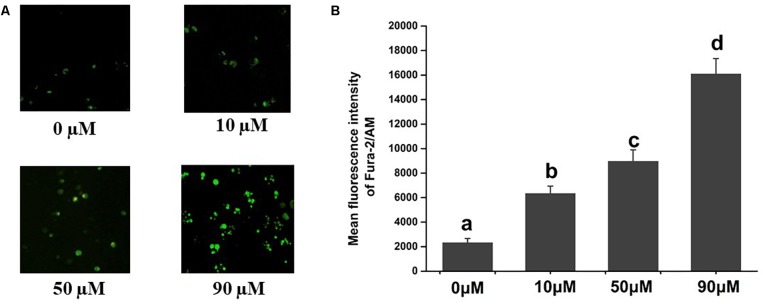
Effects of scopoletin on intracellular free calcium [Ca^2+^]i levels in insect Sf9 cells. 0, 10, 50, and 90 μM indicate Sf9 cells incubated with scopoletin at concentrations of 0, 10, 50, and 90 μM, respectively for 24 h. **(A)** The [Ca^2+^]i level was detected by Fura-2/AM fluorescence staining. Positively stained calcium is shown as green zones in the captured images under a microscope. **(B)** The bar chart indicates the mean fluorescence intensity (MFI) of Fura-2/AM in Sf9 cells. Error bars represent the standard error of the calculated mean based on three biological replicates. Different letters on the error bars indicate significant difference according to Duncan’s multiple tests (alpha = 0.05). i.e., No statistical difference between “a” and “a”; significant difference among “a,” “b,” “c,” and “d.”

Intracellular free calcium [(Ca^2+^) i] is one of the small signaling molecules regulating various biological functions in cells, including gene expression, protein synthesis, cell secretion, motility, metabolism, cell-cycle progression, and cell apoptosis (Nicotera and Orrenius, 1998). Under normal conditions, [Ca^2+^]i concentration is maintained at 10–100 nM, and intracellular Ca^2+^ homeostasis maintains the normal function of cells (Bootman et al., 2001). However, sustained Ca^2+^ release from intracellular Ca^2+^ stores, Ca^2+^ influx through receptor- or voltage-dependent Ca^2+^ channels or blockage of re-uptake can perturb Ca^2+^ homeostasis, and the increased intracellular calcium concentration [(Ca^2+^)i] induces cell apoptosis (Orrenius et al., 2003). In human or mammalian cells, the increased [Ca^2+^]i mediates the apoptosis of tumor cell induced by scopoletin in different cell types, such as T lymphoma cells (Manuele et al., 2006), PC3 cells (Liu et al., 2005), P-388 lymphocytic leukemia (Cassady et al., 1979), KB cells (Williams and Cassady, 1976), and Hepa 1c1c7 mouse hepatoma cells (Jang et al., 2003). Meanwhile, studies proved that the mode of action of scopoletin in insects was by inducing intracellular calcium overload. A significant increase in intracellular calcium level in *Spodoptera frugiperda* Sf9 cells was induced by scopoletin in a dose-dependent manner (Figure 1). Interestingly, the combination of Ca^2+^ and scopoletin can significantly improve its acaricidal activity (Hou et al., 2015). It is clear that the acaricidal mechanism of scopoletin is mainly by inducing calcium overload. However, other processes, such as MAPK signaling pathway, protein processing in endoplasmic reticulum, and fat digestion and absorption, may play a secondary role in the mode of action of scopoletin. Therefore, in this study, the molecular mechanism of calcium overload induced by scopoletin was investigated.

The main aim of the current study was to investigate the molecular mechanism of scopoletin inducing calcium overload in *T. cinnabarinus*, and attempted to provide evidence that the mode of action of the scopoletin was through the regulation of the expression of calcium signaling pathway-related genes, thus inducing calcium overload to kill mites. We conducted a comprehensive study that utilizes RNA-seq to detect changes in transcription levels. The role of candidate target genes, that is, calcium signaling pathway-related genes in the calcium overload were also investigated by RNA interference (RNAi) and a calcium reporter assay.

## Materials and Methods

### Mite Rearing

The *T. cinnabarinus* colony was originally collected from cowpeas in Beibei, Chongqing, China and maintained for more than 16 years without exposure to any pesticides (Zhang et al., 2013). Specific permission was not required for the collection because it is a harmful agricultural insect and is distributed extensively. The mites were reared on potted in cowpea seedlings (*Vigna unguiculata*) in the insectary at 25 ± 1°C, 50% ± 5% RH, and 14 h:10 h (L:D) photoperiod.

### Cell Lines and [Ca^2+^]i Assay

*Spodoptera frugiperda* Sf9 cells were cultivated at 27°C and 5% CO_2_ in 3 mL Grace’s insect cell culture medium (Gibco, United States) containing 10% fetal bovine serum (FBS), 0.3% yeast extract, 0.3% lactalbumin hydrolysate, and 0.3% peptone. Chinese hamster ovary (CHO-WTA11) cells were cultured at 37°C and 5% CO_2_ in the DMEM/F-12 medium (Invitrogen Life Technologies, Carlsbad, CA, United States) supplemented with 10% FBS, 250 ng/ml fungizone, 100 U/ml of penicillin, and 100 mg/ml of streptomycin.

In order to detect the effect of scopoletin (purity, 95%; Southwest University, Beibei, Chongqing, China) on intracellular free calcium [Ca^2+^]i levels in insect cells, the Sf9 cells were treated with diluted scopoletin at concentrations of 0, 10, 50, and 90 μM for 24 h. The [Ca^2+^]i level in the Sf9 cells was determined by Fura-2/AM fluorescence staining (Cao et al., 2016). Briefly, the harvested cells were incubated with Fura-2/AM (Beyotime, China) at final concentration of 5 μM at 30°C for 30 min. A fluorescence microscope (Carl Zeiss) was then used to observe the cells. The [Ca^2+^]i level was represented by the mean fluorescence intensity (MFI) after the captured images were analyzed using Image-Pro Plus software (Media Cybernetics).

### Bioassays and Scopoletin Treatment

The FAO-recommended slip-dip method was used to measure scopoletin toxicity against adult female *T. cinnabarinus* (Busvine, 1980). The details of the bioassay procedure were described by Zhang et al. (2013). In brief, 30 adult female individuals (3–5 days old) were placed on their backs on double-sided tape on glass. Then, the mites were dipped into each test solution for 5 s. Each dose (2, 1, 0.5, 0.25, 0.125, and 0.0625 g/ml) was performed in three replicates. Sterile distilled water with 0.1% (v/v) Tween-80 and 3% (v/v) acetone was designated as the control treatment. The mites were observed under an anatomical microscope after 48 h of rearing under controlled growth conditions as described above. Mites that exhibited immobility or irregularly trembling legs were considered dead. The lethal concentrations for subsequent experiments were determined on the basis of log-probit analysis of concentration–mortality data.

For the analysis of the transcriptome changes in *T. cinnabarinus* treated with scopoletin or the solvent, scopoletin was dissolved in sterile distilled water containing 0.1% Tween 80 and 3% acetone to a final concentration of 0.938 mg/mL, the median lethal concentration (LC_50_) of scopoletin against *T. cinnabarinus*. For the scopoletin exposure experiment, we adopted a slightly modified version of the leaf-disk dipping method described by Michel et al. (2010). More than 200 female adults (3–5 days old) were transferred to three freshly potted cowpea leaves in a small petri dish with water. Each detached cowpea leaf was dipped for 5 s in the test solution at the concentration indicated above. When the liquid dried around the mites, the insects were returned to the conditions as above. Then, sterile distilled water with 0.1% Tween-80 and 3% acetone was used as the solvent control. Three petri dishes from one independent experiment comprised a replicate and two biological replicates used for RNA purification and library preparation. After 24 and 48 h intervals, only the surviving female adult mites from the treated and control groups were collected and frozen at -80°C for RNA extraction.

### RNA Extraction, Library Preparation, and Sequencing

The total RNA of each sample was extracted using the RNeasy^®^ plus Micro Kit (Tiangen, Beijing, China) following the manufacturer’s instructions. For checking the RNA quantity, the absorbance at 260 nm and absorbance ratio of OD_260/280_ were measured using a Nanovue UV-Vis spectrophotometer (GE Healthcare, Fairfield, CT, United States). RNA integrity was further confirmed by 1% agarose gel electrophoresis.

The polyA mRNA was enriched from the total RNA using the Dynabeads mRNA Purification Kit (Invitrogen) and digested into short fragments (∼130 bp) with First-Strand Buffer (Invitrogen) at the appropriate temperature. The short fragments served as templates to synthesize first-strand cDNA with random hexamer primers, First-Strand Master Mix, and Super Script II reverse transcriptase (Invitrogen). Then, second-strand cDNA was synthesized using the Second-Strand Master Mix. After adenylation of the 3’ ends of DNA fragments, the sequencing adaptors were ligated. AMPure XP beads were used to purify the short fragments; these cDNA were eluted in EB buffer, followed by polymerase chain reaction (PCR) amplification. An Agilent 2100 Bioanalyzer checked the quality of the library and the concentration of cDNA. The prepared libraries were sequenced on the Ion Proton platform (BGI, Shenzhen, China) using the sequencing strategy of single-end 150 bp.

### Processing and Mapping of RNA-Seq Data

Primary sequencing data was first subjected to quality control. These data were produced by Ion Proton and were called raw reads. For filtering the raw reads, low-quality and adapter sequences were removed. The Q20, Q30, and GC contents of the filtered reads were calculated and checked. All obtained high-quality and clean reads were mapped against the reference genome of *Tetranychus urticae* with T-Map (Version 3.4.1, parametermapall-a2-n8-v-Y-u-o1stage1map4) ^1^. Mismatches of three or less than three bases for each read (mean length = 150 bp) were allowed in the mapping. The unique and non-unique mapped reads were used for mapping scale calculation. The read per kilobase per million mapped reads (RPKM) of each gene was calculated using the following formula: RPKM = total exon reads/mapped reads in million × exon lengths in kb. The RPKM value was used as expression levels for differential expression analysis.

### Differential Expression Analysis

To identify differentially expressed genes between different treatments, we used a rigorous algorithm as previously described (Audic and Claverie, 1997). The false discovery rate (FDR) was calculated to determine the threshold *p*-value in multiple tests. In this study, a threshold FDR ≤ 0.001 and an absolute value of Log_2_Ratio ≥ 1 were used to determine the significance of gene expression differences (Benjamini and Yekutieli, 2001). For depth analysis of differentially expressed genes, cluster analysis was performed using Cluster software and Java Treeview software. We then mapped, all differentially expressed genes to terms in the Kyoto Encyclopedia of Genes and Genomes (KEGG) database and the gene orthology (GO) database for annotation.

### Total RNA Extraction, cDNA Synthesis, and TcGPCR, TcBAG, and TcGUK Cloning

Total RNA was extracted from 300 adult (3–5 days old) *T. cinnabarinus* females. Extraction was performed as described in the above. Reverse transcription was performed with PrimeScript^®^ First Strand cDNA Synthesis Kit (Takara, Dalian, China). Synthesized cDNA was stored at -20°C. To obtain the full-length *TcGPCR*, *TcBAG*, and *TcGUK*, we designed and synthesized specific primers (Supplementary Table S1) based on complete genomic sequences from the sister species *T. urticae*^2^. Specific PCR reactions were performed in a C1000^TM^ Thermal Cycler (BIO-RAD, Hercules, CA, United States). PCR reactions were performed with a 25 μL reaction volume with 2.5 μL 10 × PCR buffer (Mg^2+^ free), 2.0 μL dNTPs (2.5 mM), 2.5 μL MgCl_2_ (25 mM), 1 μL cDNA templates, 1 μL of each primer (10 mM), 0.2 μL rTaq^TM^ polymerase (TaKaRa), and 14.8 μL double-distilled H_2_O (ddH_2_O). The PCR program was 94°C for 3 min, followed by 35 cycles of 94°C for 30 s, 48°C to 60°C (based on the primer annealing temperature) for 30 s, 72°C extension for 1 min to 2 min (based on the predicted length of the amplified products), and a final extension for 10 min at 72°C. The amplified PCR fragments were gel-purified using the Gel Extraction Mini Kit (Tiangen, Beijing, China), ligated into pMD^TM^ 19-T Vector (Takara, Dalian, China), and then transformed into *Escherichia coli* Trans5α-competent cells (Tiangen, Beijing, China). Recombinant plasmids were sequenced at the Beijing Genomics Institute (BGI, Beijing, China).

### Gene Characterization and Phylogenetic Analysis

The nucleotide sequences of *TcGPCR*, *TcBAG*, and *TcGUK* nucleotide sequences were edited with DNAMAN 5.2.2. The deduced amino acid sequences of the GPCR, BAG, and GUK genes were aligned with ClustalW program (Hill et al., 2004; Bansal et al., 2011). The molecular weight and isoelectric point (pI) of the proteins were calculated by ExPASy Proteomics Server^3^ (Bairoch, 1993). The signal peptide was predicted using SignalP 4.1^4^ (Bendtsen et al., 2004), and the transmembrane region was analyzed using the TMHMM Server (v.2.0)^5^ (Krogh et al., 2001). The *N*-glycosylation sites were predicted by the NetNGlyc 1.0 Server^6^ (Gupta et al., 1997). The phylogenetic tree was constructed with MEGA 5.0 via the neighbor-joining method with 1000 bootstrap replicates (Tamura et al., 2011).

### dsRNA Synthesis, dsRNA Feeding, and Knockdown *TcGPCR*, *TcBAG*, and *TcGUK* by RNAi

A set of T7 RNA polymerase promoter primers (Supplementary Table S1) were designed to amplify 160–600 bp lengths of the target genes to generate PCR products for *in vitro* transcription and dsRNA production (Supplementary Table S1). *TcGPCR*, *TcBAG*, *TcGUK* and the Green Fluorescent Protein (*GFP*) (ACY56286) gene were amplified by PCR. The PCR program was as described above. The recombinant plasmids were used as a template. The *GFP* gene was used as a negative control. The amplified segments were gel-purified and used in the TranscriptAid T7 High Yield Transcription Kit (Thermo Fisher Scientific, Lithuania, Europe). The dsRNAs were further purified with GeneJET RNA Purification Kit (Thermo Fisher Scientific, Lithuania, Europe). The size of the dsRNA products was determined by 1% agarose gel electrophoresis. The concentration of dsRNAs was determined with a spectrophotometer. dsRNAs were stored at -70°C. The systemic delivery of dsRNA via leaf-disk feeding was used to knock down *TcGPCR, TcBAG*, and *TcGUK* expressions, respectively. In brief, cowpea leaves were cut to a feeding arena (2.0 cm diameter) and dehydrated via incubation at 60°C for 3–5 min. Then, the leaves were treated with DEPC–water, dsRNA–*GFP*, or dsRNA–(*TcGPCR, TcBAG*, and *TcGUK*, respectively) (1000 ng/μL) for 3–4 h at room temperature. After complete absorption of the liquids, the leaves were placed on wet filter paper. Then, the leaf disks were placed on water-saturated sponges. Thirty female adults (3–5 days old and starved for 24 h) were placed on each dsRNA-permeated leaf disk. Then, the leaf disks were placed upside down on petri dishes (7 cm in diameter) to prevent mites from escaping. The dsRNA-treated leaf disks, which were infested by *T. cinnabarinus*, were placed under controlled growth conditions as described above. Finally, the mites were collected for subsequent experiments at 48 h post-feeding.

### Quantitative Real-Time PCR (qPCR)

To verify the differential expressions of some genes generated by the abovementioned parameters by qPCR, we randomly selected 15 genes from significantly differentially expressed genes. To detect *TcGPCR*, *TcBAG*, and *TcGUK* expressions throughout the different life stages of the mites, approximately 2000 eggs, 1500 larvae, 800 nymphs, and 200 adults were collected per sample with three replicates. To quantify *TcGPCR*, *TcBAG* and *TcGUK* expressions at 24 and 48 h, in response to different concentrations of scopoletin exposure, we collected 200 female adults per sample with three replicates. For examining the effect of scopoletin exposure on *TcGPCR*, *TcBAG*, and *TcGUK* expressions, female adults were treated with scopoletin, with 0.1% (v/v) Tween-80 and 3% (v/v) acetone as the surfactants. As in the slip-dip assay, the LC_10_, LC_30_, and LC_50_ of scopoletin corresponded to 0.099, 0.374, and 0.938 mg/mL, respectively. For the scopoletin exposure experiment, we adopted the leaf-disk dipping method described above and the detailed bioassay procedure that was described by Michel et al. (2010). Each experiment was replicated for a minimum of three times and used independent biological samples. For examining the effectiveness of RNAi, approximately 200 female adult mites were collected per sample at 48 h post-dsRNA feeding. Three replicated samples were prepared. The specific primers used for qPCR were designed by Primer 3.0^7^ (Supplementary Table S1; Misener and Krawetz, 2000). *RPS18* (FJ608659) was used as the stable reference gene for all qPCR assays (Supplementary Table S1; Sun et al., 2010). qPCR was conducted with a Mx3000P thermal cycler (Agilent Technologies, Inc., Wilmington, NC, United States) with 20 μL reaction mixtures that contained 1 μL cDNA template (200 ng/μL), 10 μL iQ^TM^ SYBR^®^ Green Supermix (BIO-RAD, Hercules, CA, United States), 1 μL of each gene-specific primer (0.2 mM), and 7 μL ddH_2_O. The optimized qPCR protocol used for amplification was 95°C for 2 min, followed by 40 cycles of denaturation at 95°C for 15 s, 60°C for 30 s, and elongation at 72°C for 30 s. Melt curve analyses (from 60 to 95°C) were included to ensure the consistency of the amplified products. The quantification of expression level was analyzed using the 2^-ΔΔ^*^Ct^* method (Livak and Schmittgen, 2001).

### Susceptibility Test of *T. cinnabarinus* to Scopoletin After RNAi of *TcGPCR, TcBAG*, and *TcGUK*

Lethal doses of scopoletin (LC_50_ of the scopoletin) were applied in the bioassays. We adopted the slip-dip method described above and the detailed bioassay procedure that was described by Ding et al. (2013). The LC_50_ values of scopoletin were used as diagnostic doses to compare the changes in susceptibility to acaricide in *T. cinnabarinus* at 48 h post-feeding of dsRNA- (*TcGPCR, TcBAG*, and *TcGUK*, respectively).

### Heterologous Expression and Functional Assay

To construct the plasmid for transient expression, the ORF of the *TcGPCR* was inserted into the expression vector pcDNA3.1(+) with the restriction enzyme BamHI and XbaI (TaKaRa). The sequences of the inserts were confirmed by sequencing (BGI) prior to heterologous expression. High-quality plasmid DNA prepared using the EndoFree Maxi Plasmid Kit (Tiangen) was employed for transient transfection. CHO-WTA11 cells supplemented with aequorin and Gα16 were used for heterologous expression. The cells were collected 30 h later and pre-incubated with the coelenterazine (Invitrogen) for the functional assay according to the published protocols (Aikins et al., 2008; Jiang et al., 2014). Luminescence caused by intracellular calcium mobilization was measured using a TriStar^2^ LB 942 Multimode Reader (Berthold Technologies, Bad Wildbad, Germany). Ten-fold serial dilutions of the scopoletion were used for the treatment of the cells. Based on luminescence values, a concentration-response curve of the receptor to the scopoletin was developed using logistic fitting in Origin 8.6 (OriginLab^8^). The experiments were conducted in three biological replicates.

### Statistical Analysis

The MFI of Fura-2/AM in Sf9 cells, the differences in the expression levels of *TcGPCR*, *TcBAG*, and *TcGUK* during four developmental stages, RNAi knockdown efficiencies, and mortality rates were analyzed by one-way analysis of variance (ANOVA), followed by Duncan’s multiple tests in SPSS (v.16.0, SPSS Inc., Chicago, IL, United States) at a alpha = 0.05.

## Results

### Analysis of Acaricidal Toxicity

Table 1 presents the median lethal concentration values (LC_50_) calculated for scopoletin against adult female *T. cinnabarinus*. The estimated LC_50_ values of scopoletin was 0.938 mg/mL. The LC_50_ of scopoletin indicated its potential as an acaricidal compound against *T. cinnabarinus*.

**Table 1:** Toxicity of scopoletin against adult females of *T. cinnabarinus* after 48 h exposure time.

Acaricide	N	LC_50_ (mg.mL-^1^)^a^ 95%CI^b^	Slope (±SE)	χ*^2^*^c^	*P*
scopoletin	540	0.938 (0.576∼2.292)	1.314 ± 0.15	6.321	0.097

*^a^LC_50_, median lethal concentration. ^b^CI^,^ 95% confidence interval. ^c^Chi-square testing linearity, P < 0.05.*

### Effects of Scopoletin on Intracellular Free Calcium [Ca^2+^]i Levels in Sf9 Cells

The images of Fura-2/AM staining of the Sf9 cells are shown in Figure 1. As a calcium indicator, the intensities of Fura-2/AM staining were used to determine the [Ca^2+^]i concentration. Treatment with scopoletin significantly elevated the [Ca^2+^]i levels in insect Sf9 cells in a concentration-dependent manner (Figure 1).

### RNA-Seq Data Analysis

For investigating the transcriptional changes in *T. cinnabarinus* after scopoletin treatment, the purified mRNA from scopoletin- or solvent-treated mites were sequenced on the Proton platform. After the removal of duplicate sequences, adaptor sequences, and low-quality reads, 52,496,305 clean sequence reads were generated from scopoletin-treated mites, and 52,286,859 clean sequence reads were generated from solvent-treated mites (Supplementary Table S2). All sequencing data were submitted to the GEO web site^9^ with the accession number GSE92959. The whole genome sequence of *T. cinnabarinus* is still unavailable; therefore, the genomic information of the sister species *T. urticae* was used as the reference genome for map reading. More than 80% of these reads could be successfully mapped to the reference genome, indicating the overall good quality of RNA-seq, as well as the close genetic relationship between *T. cinnabarinus* and *T. urticae*. When the total read numbers approached 5 million per sample, sequencing saturation analysis showed that the number of detected genes tends to be saturated, and the amount of sequencing data can be determined to meet the requirements (Supplementary Figure S2). Each of our libraries produced up to 10 million reads, indicating that the depth of sequencing was sufficient to cover most of the transcripts of this organism.

### Differential Gene Expression Between Scopoletin- and Solvent-Treated Mites

**FIGURE 2:**
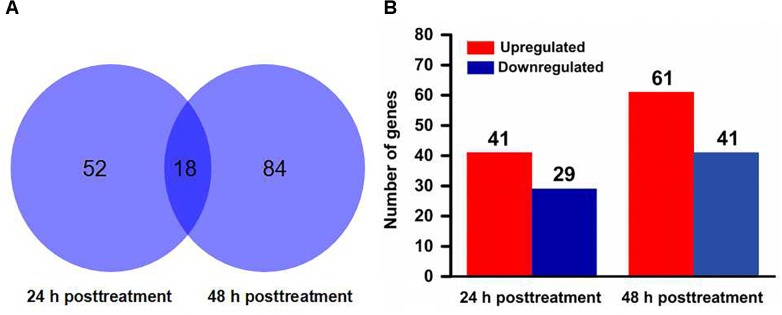
Distribution of differentially expressed genes in *Tetranychus cinnabarinus* in response to scopoletin. **(A)** Venn diagram showing the total number of differentially expressed genes at 24 and 48 h posttreatment, and the number of overlapped genes between two time points. **(B)** The number of up-regulated and down-regulated genes at 24 and 48 h after scopoletin treatment.

A total of 18,414 protein coding gene models in the *T. urticae* genome database (Grbiæ et al., 2011). More than 14,000 genes were detected for expression in each sample by mapping all clean reads to the reference genome (Supplementary Table S2). According to RPKM values, 70 and 102 genes were identified as significantly differentially expressed genes between scopoletin- and solvent-treated mites for 24 and 48 h, respectively (Supplementary Table S3, S4). Most genes were time-specific except for 18 genes that were shared by two time points, as found by comparing the differentially expressed genes at two time points (Figure 2A and Supplementary Table S4). Among the significantly differentially expressed genes, 41 genes were upregulated and 29 genes were downregulated by scopoletin treatment for 24 h, and 61 genes were upregulated and 41 genes were downregulated by scopoletin treatment for 48 h (Figure 2B). The log_2_ fold change was from -12.8 to 13.4. At 24 and 48 h post-treatment, the number of upregulated significantly differentially expressed genes was consistently higher than that of downregulated genes. Moreover, the number of significantly differentially expressed genes in the treatment at 48 h was markedly higher than that at 24 h by scopoletin. This difference suggested that certain genes are regulated by scopoletin with the change in scopoletin treatment time and that the significantly differentially expressed genes may play a key role in the acaricidal mechanism of scopoletin against *T. cinnabarinus*.

### GO Enrichment and KEGG Pathway Analysis of Differentially Expressed Genes

**FIGURE 3:**
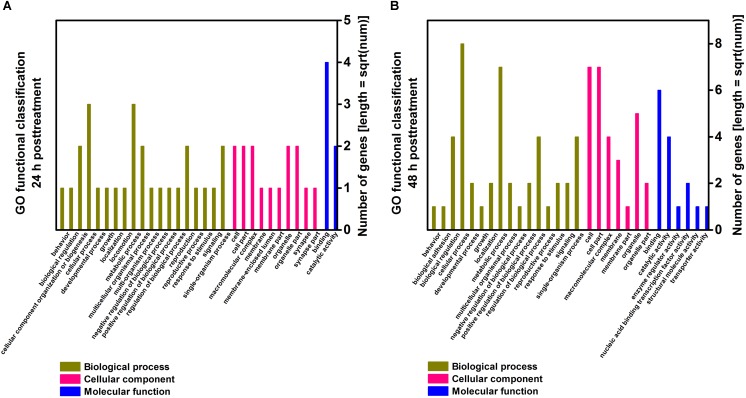
Gene ontology (GO) enrichment analysis of differentially expressed genes in *T. cinnabarinus* after scopoletin or solvent treatment. **(A)** Functional categories of differentially expressed genes at 24 h posttreatment. **(B)** Functional categories of differentially expressed genes at 48 h posttreatment. Three main categories, biological process, cellular component and molecular function, are summarized.

To understand the molecular function of genes involved in the response of *T. cinnabarinus* to scopoletin treatment, we used GO database assignments to classify the functions of the predicted genes by mapping all the differentially expressed genes to terms into the GO database and comparing them with the whole reference genome background (Figure 3). Based on three GO classes, namely, biological processes, cellular components, and molecular functions, the differentially expressed genes from 24 h post-treatment were categorized into 31 GO subgroups (Figure 3A) and differentially expressed genes from 48 h post-treatment were categorized into 29 GO subgroups (Figure 3B). In the biological processes category, the “cellular processes” category was prevalent, followed by “metabolic process” throughout the GO classification. At 24 h post-treatment, one enriched term (mitotic nuclear division) presented a proportion of 50%. At 48 h post-treatment, three enriched terms, including ion binding (33.3%), regulation of signal transduction (25%), and nervous system development (25%), were observed. In the cellular components category, the most highly represented subgroups were “cell” and “cell part.” In the molecular functions classification, the major subgroups were “catalytic activity” and “binding.” Interestingly, the major categorized subgroups were relatively similar for 24 and 48 h post-treatment, thereby indicating a similar response pattern of mites toward scopoletin treatment at different time points.

**FIGURE 4:**
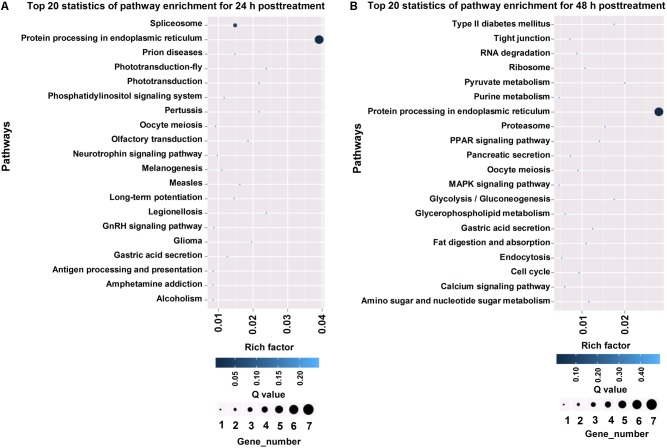
KEGG pathway analysis of differentially expressed genes in *T. cinnabarinus* in response to scpoletin. **(A)** Top 20 enriched KEGG pathways of differentially expressed genes at 24 h posttreatment. **(B)** Top 20 enriched KEGG pathways of differentially expressed genes at 48 h posttreatment. Rich factor is defined by the ratio of the number of differentially expressed genes enriched in the pathway and the number of all genes enriched in the same pathway.

To annotate the differentially expressed genes, we aligned the genes into the KEGG database for functional prediction and classification (Figure 4). Among the top 20 pathways at 24 and 48 h post-treatment, “protein processing in endoplasmic reticulum” represented the major biochemical pathway (Figure 4). Pathways, such as phosphatidylinositol signaling system, spliceosome, and gastric acid secretion, were significantly enriched at 24 h post-treatment (Figure 4A), whereas calcium signaling pathway, MAPK signaling pathway, and fat digestion and absorption were well represented at 48 h post-treatment (Figure 4B).

### Identification of Candidate Genes Involved in Mite Detoxification and Acaricidal Mechanism

On the basis of the previous analysis on differentially expressed genes, we manually selected candidate genes associated with mite detoxification and acaricidal mechanism, such as cell proliferation, substance transportation, cell apoptosis, detoxification, and metabolism (Tables 2, 3). The gene products of these candidates could be classified into several categories, such as signal transduction protein, apoptosis protein, energy metabolism protein, and channel protein, according to their biological functions. Genes involved in signal transduction were guanylate kinase, G-protein coupled neuropeptide receptor, and glycerol-3-phosphate dehydrogenase. We identified senescence-associated protein, Bcl-2 protein, RAB5-interacting protein as cell apoptosis-related products. Moreover, C4-dicarboxylate-binding protein was detected as an energy metabolism-related protein. Among the selected candidate genes, genes associated with signal transduction and cell apoptosis were dominant. Interestingly, several candidates, such as the C4-dicarboxylate-binding protein, were upregulated at 24 h post-treatment and downregulated at 48 h post-treatment. However, except for its acaricidal activity, scopoletin is widely used as a medicine for human beings. Surprisingly, genes with similar functional annotations to the targets of scopoletin were found in our study. For example, Bcl-2 protein, GUK, and RAB5-interacting protein were differentially expressed in mites treated with scopoletin.

**Table 2:** Selected genes involved in mite detoxification and acaricide metabolism at 24 h posttreatment.

Gene ID	Description	RPKM		Log_2_ fold change
		CK	Scopoletin	
tetur11g05740	Lipase	11.6255	1.4205	-3.03
tetur32g01740	Bcl-2 protein	8.4983	1.6847	-2.33
tetur03g04050	Thioredoxin-like protein 4A	15.6866	5.7149	-1.46
tetur06g00140	Senescence-associated protein	242.9565	107.2315	-1.18
tetur07g05920	Guanylate kinase	46.8228	22.8164	-1.04
tetur11g01680	Heat shock protein 70	128.5005	63.8484	-1.01
tetur21g00510	RAB5-interacting protein	16.3942	52.5307	1.68
tetur02g02050	C4-dicarboxylate-binding protein	1.7990	7.9400	2.14
tetur01g05810	Exostosin-1	0.0100	18.0346	10.82

**Table 3:** Selected genes involved in mite detoxification and acaricide metabolism at 48 h posttreatment.

Gene ID	Description	RPKM		Log_2_ fold change
		CK	Scopoletin	
tetur07g05920	Guanylate kinase	71.2255	0.0100	-12.80
tetur02g02050	C4-dicarboxylate-binding protein	6.6234	0.0100	-9.37
tetur201g00020	Prohibitin 2	8.6853	0.5558	-3.97
tetur08g07000	AMP-dependent synthetase and ligase	9.9772	0.8585	-3.54
tetur02g05380	Scaffold protein	9.1194	0.8489	-3.43
tetur02g02600	26S protease regulatory subunit 6A	13.2758	1.6563	-3.00
tetur05g04350	Glycosyltransferase subunit 4	32.6193	4.6704	-2.80
tetur06g00140	Senescence-associated protein	266.4575	38.5752	-2.79
tetur651g00010	Ribosomal protein S12	21.0008	4.4955	-2.22
tetur39g00730	Vitellogenin1	17.4034	4.1871	-2.06
tetur23g01300	Glycerol-3-phosphate dehydrogenase	9.9892	2.5329	-1.98
tetur02g12070	Tanscription factor SOX-2	17.6862	6.0849	-1.54
tetur15g01820	DM DNA binding domain	29.7538	12.7198	-1.23
tetur11g01430	Similar to Negative elongation factor E CG5994-PA	45.0860	94.5915	1.07
tetur01g05420	BmGATA-beta:Transcription factor BCFI	3.9215	15.9409	2.02
tetur12g04490	DEAD-box ATP dependent DNA helicase	2.5244	11.3534	2.17
tetur08g06300	ADP-ribosylation factor-like protein 6	6.2265	30.8088	2.31
tetur02g03830	G-protein coupled neuropeptide receptor	3.0684	18.2442	2.57
tetur21g01620	Similar to GA21569-PA	0.1296	6.2176	5.58
tetur17g02410	ADP-ribosylation factor	0.0914	4.5619	5.64
tetur01g05810	Exostosin-1	0.0100	22.5082	11.14
tetur04g07080	Alpha-D-phosphohexomutase	0.0100	109.2025	13.41

### Validation of RNA-Seq Data by RT-qPCR

**FIGURE 5:**
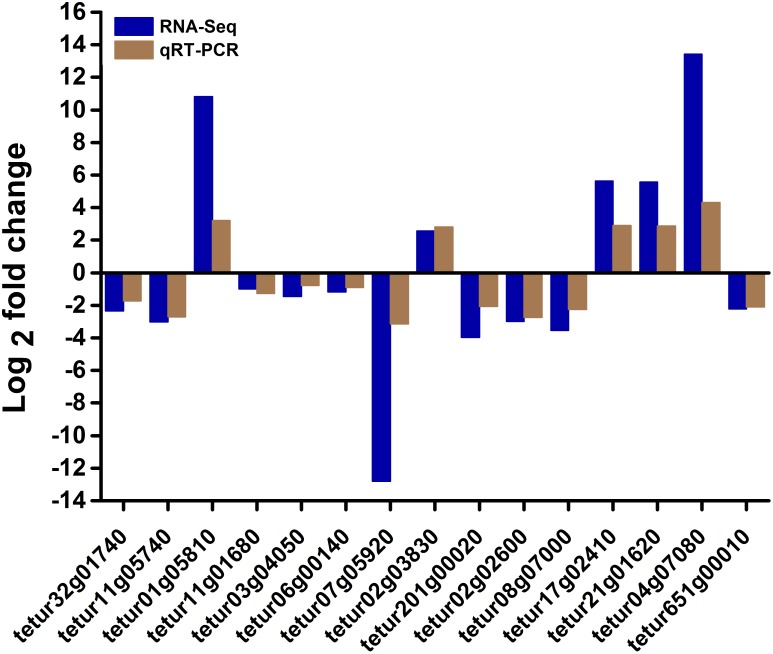
RT-qPCR validation of some differentially expressed genes in RNA-seq. The relative expression levels of fifteen differentially expressed genes in scopoletin- or solvent-treated *T. cinnabarinus* were determined by RT-qPCR. *RPS18* was used as the reference gene to normalize the gene expression using the ΔΔCq method. The y-axis indicates the log_2_ fold change of each gene in RT-qPCR and RNA-seq.

To confirm the RNA-seq results, we selected 5 upregulated genes and 10 downregulated genes from the differentially expressed genes involved in mite detoxification and acaricide mechanism either at 24 or 48 h post-treatment. These genes were used for quantitative reverse transcription PCR (RT-qPCR) analysis. The results of RT-qPCR showed that all tested genes presented a similar differential expression trend compared with the RNA-seq data (Figure 5). For example, the ADP-ribosylation factor tetur17g02410 and the exostosin-1 tetur01g05810 were upregulated by 5.6 and 10.8 log_2_ fold changes, respectively, in the RNA-seq and by 2.9 and 3.2 log_2_ fold changes, respectively, in RT-qPCR. Moreover, the results of RT-qPCR showed that the log_2_ fold change of the tested genes did not perfectly match that in the RNA-seq possibly because of calculation and sequencing bias. However, the RT-qPCR results almost validated the upregulation and downregulation directions obtained from RNA-seq results.

### cDNA Cloning and Characterization of *TcGPCR, TcBAG*, and *TcGUK*

**FIGURE 6:**
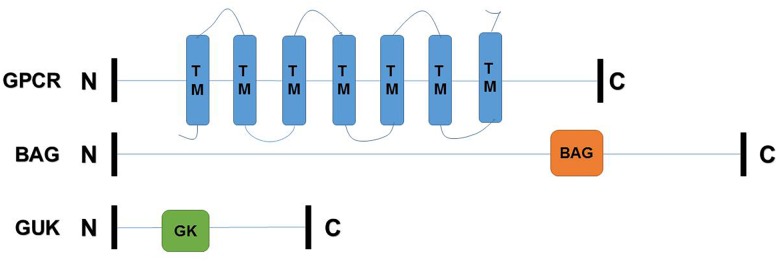
Schematic drawing of *TcGPCR, TcBAG, TcGUK*. C, C-terminal region; N, N-terminal region; TM, transmembrane helices; BAG, BAG domain; GK, guanylate kinase-like domain.

The deduced amino acid sequences and full-length cDNAs of *TcGPCR, TcGUK*, and *TcBAG*, which contained open reading frames (ORFs), were deposited in GenBank under the following accession numbers: KY660538 (*TcGPCR*), KY660539 (*TcBAG*), and KY660540 (*TcGUK*). Supplementary Table S5 presents the lengths of the deduced amino acid sequences, predicted protein molecular weights, and theoretical isoelectric points. *TcGPCR* was predicted to possess seven transmembrane (TM) helices, as shown in Figure 6. These regions were reported as rhodopsin-like GPCR (GPCRA) common structure frameworks (Monaci et al., 1990). The GPCRA represents a widespread protein family that includes hormones, neurotransmitters, and light receptors involved in signal transmission (Casey and Gilman, 1988). *TcBAG* was predicted to include a BAG domain that has anti-apoptotic activity and increases the anti-cell death function of Bcl-2 induced by various stimuli (Figure 6; Doong et al., 2002). *TcGUK* is predicted to possess a guanylate kinase-like domain (GK) whose function is to mediate the interaction of protein molecules, which is related to cell adhesion and orientation of mitotic spindle (Figure 6; Momand et al., 2000; Von et al., 2003; Yang et al., 2004).

### Phylogenetic Analysis of *TcGPCR*, *TcBAG*, and *TcGUK*

**FIGURE 7:**
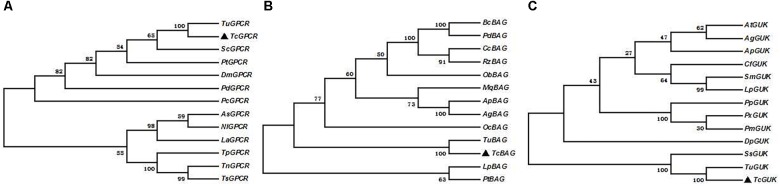
Phylogenic analysis of *TcGPCRs, TcBAGs* and *TcGUKs*, respectively. Maximum likelihood tree constructed by MEGA 5.0. Phylogeny testing was conducted via the bootstrap method with 1000 replications. **A**, **B**, and **C** were *TcGPCRs, TcBAGs*, and *TcGUKs*, respectively.

Phylogenetic analysis was performed by MEGA 5.0 with the maximum-likelihood method on the basis of the deduced amino acid sequences of *TcGPCR*, *TcBAG*, and *TcGUK*, as well as other known GPCR, BAG, and GUK proteins, including orthologs from arachnids and insects. All GPCR, BAG, and GUK sequences, which possess complete ORFs, were obtained from the *T. urticae* genome and the National Center for Biotechnology Information (Bethesda, MD)^10^ (Supplementary Table S6). The result showed that *TcGPCR*, *TcBAG*, and *TcGUK* share the highest sequence similarity with the GPCR, BAG, and GUK of *T. urticae* (*TuGPCR*, *TuBAG*, and *TuGUK*), respectively (Figure 7), suggesting evolutionary relatedness and possibly similar physiological functions that exist between *TcGPCR* and *TuGPCR*, between *TcBAG* and *TuBAG*, and between *TcGUK* and *TuGUK*.

### Expression Patterns of *TcGPCR, TcBAG*, and *TcGUK* in Different Developmental Stages and Scopoletin Treatment

**FIGURE 8:**
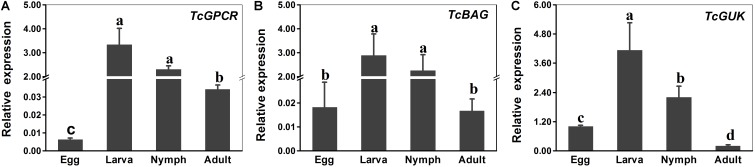
RT-qPCR evaluation of the developmentally specific expression patterns of GPCR, BAG and GUK genes in *T. cinnabarinus*, respectively. **(A)** RT-qPCR analysis of *TcGPCR* expression in different life stages. **(B)** RT-qPCR analysis of *TcBAG* expression in different life stages. **(C)** RT-qPCR analysis of *TcGUK* expression in different life stages. The following life stages were analyzed: egg, larvae, nymph, and adult. Error bars represent the standard error of the calculated mean based on three biological replicates. Different letters on the error bars indicate significant difference according to Duncan’s multiple tests (alpha = 0.05). i.e., No statistical difference between “a” and “a”; significant difference among “a,” “b,” “c,” and “d.” *RPS18* was used as the reference gene.

The expression levels of *TcGPCR*, *TcBAG*, and *TcGUK* genes during different developmental stages (egg, larva, nymph, and female adult) and upon acaricide treatment were evaluated via qPCR. The results showed that the calcium channel-related genes (*TcGPCR*, *TcBAG*, and *TcGUK*) were expressed throughout all life stages, which suggested that *TcGPCR*, *TcBAG*, and *TcGUK* are involved in biological processes throughout developmental and growth stages. Specifically, the calcium channel-related genes (*TcGPCR*, *TcBAG*, and *TcGUK*) were significantly highly expressed during the larval and nymphal stages compared with other developmental stages (Figure 8). The mRNA expression levels of *TcGPCR*, *TcBAG*, and *TcGUK* in larva and nymph were approximately 139-, 147-, and 5-fold higher than those in eggs and adults, respectively (Figure 8).

**FIGURE 9:**
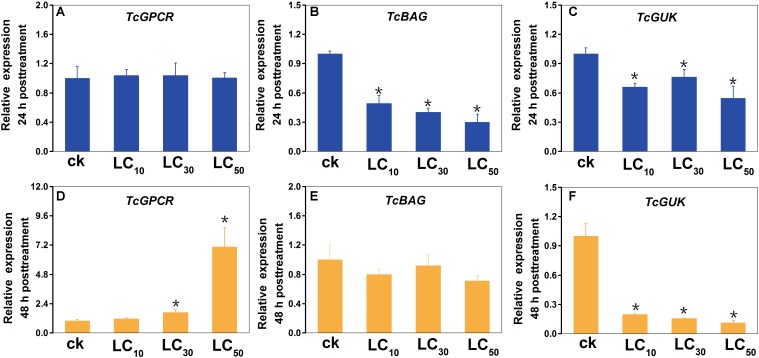
Expression profiles of *TcGPCR, TcBAG*, and *TcGUK* transcripts after scopoletin treatment for 24 h and 48 h at three different concentrations, respectively. Relative expression of the *TcGPCR* **(A,D)**, *TcBAG* **(B,E)** and *TcGUK* **(C,F)** of *T. cinnabarinus* exposed to 0.099, 0.374, and 0.938 mg/L scopoletin (LC_10_, LC_30,_ and LC_50_) in 0.1% (v/v) Tween-80 and 3% (v/v) acetone at the adult stage for 24 and 48 h using a slip-dip bioassay were analyzed using RT-qPCR, respectively. Error bars represent the standard error of the calculated mean based on three biological replicates. Water containing 0.1% (v/v) Tween-80 and 3% (v/v) acetone was used as the control treatment (CK). An asterisk (^∗^) on the error bar indicates a significant difference between the treatment and group (CK) according to *t*-tests, ^∗^*p* < 0.05. *RPS18* was used as the reference gene.

The results of the scopoletin treatment experiment showed that, compared with the control, the BAG (at 24 h post-treatment) and GUK (at 24 and 48 h post-treatment) genes were downregulated, and the GPCR gene (at 48 h post-treatment) was upregulated (Figure 9). Statistical analysis suggested that, compared with the control (CK), at LC_50_, LC_30_, and LC_10_ doses of scopoletin, the relative expression levels of *TcBAG* were 3.4-, 2.5-, and 2.0-fold lower at 24 h post treatment; the relative expression levels of *TcGUK* were 1.8-, 1.3-, and 2.4-fold lower at 24 h post-treatment and were 8.9-, 6.4-, and 5.1-fold lower at 48 h post-treatment; and the relative expression levels of *TcGPCR* were 7.1-, 1.7-, and 1.1-fold higher at 48 h post-treatment, respectively. However, the relative expression levels of *TcGPCR* after 24 h of scopoletin treatment and *TcBAG* after 48 h of scopoletin treatment were not significantly different compared with the control at three different concentrations.

### RNAi via dsRNA Knockdown

**FIGURE 10:**
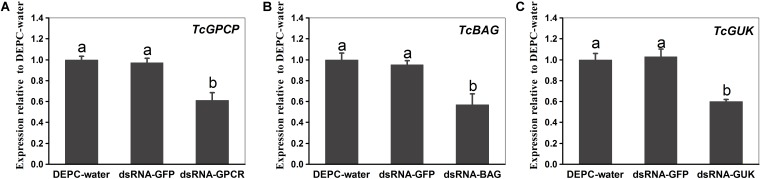
Quantitative PCR expression analysis of *TcGPCR, TcBAG*, and *TcGUK* after RNAi at 48 h post-dsRNA feeding, relative to expression levels after DEPC-water treatment, respectively. **(A)** Expression levels of *TcGPCR*. **(B)** Expression levels of *TcBAG*. **(C)** Expression levels of *TcGUK*. Different letters on the error bars indicate significant difference according to Duncan’s multiple tests (alpha = 0.05). i.e., No statistical difference between “a” and “a”; significant difference between “a” and “b.”

For investigating the transcript knockdown efficiency of the calcium channel-related genes (*TcGPCR*, *TcBAG*, and *TcGUK*) expression, relative mRNA expression levels were measured via qPCR at 48 h post-dsRNA feeding. The results showed that the transcript levels of *TcGPCR*, *TcBAG*, and *TcGUK* significantly decreased to 38.63, 43.12, and 40.13% after feeding of dsRNA*-TcGPCR*, dsRNA*-TcBAG*, and dsRNA*-TcGUK* compared with feeding of DEPC–water or dsRNA-*GFP*, respectively (Figure 10). No significant transcript efficiency difference exists between the two controls (water and dsGFP) (Figure 10). These results revealed that the *TcGPCR*, *TcBAG*, and *TcGUK* transcripts were successfully knocked down by RNAi in *T. cinnabarinus*.

### Susceptibility Test of *T. cinnabarinus* to Scopoletin After RNAi of *TcGPCR*, *TcBAG*, and *TcGUK*

**FIGURE 11:**
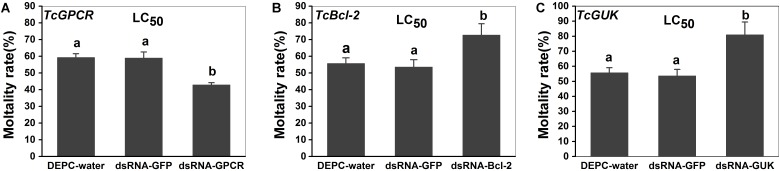
Knockdown of *TcGPCR* expression reduced susceptibility to scopoletin, and knockdown of *TcBAG* and *TcGUK* expression increased susceptibility to scopoletin in mites, respectively. **(A)** Mortality of *TcGPCR*-silenced *T. cinnabarinus* to scopoletin at LC_50_. **(B)** Mortality of *TcBAG*-silenced *T. cinnabarinus* to scopoletin at LC_50_. **(C)** Mortality of *TcGUK*-silenced *T. cinnabarinus* to scopoletin at LC_50_. Error bars represent the standard error of the calculated mean based on three biological replicates. Different letters on the error bars indicate significant difference according to Duncan’s multiple tests (alpha = 0.05). i.e., No statistical difference between “a” and “a”; significant difference between “a” and “b.”

The susceptibilities to scopoletin at 48 h after feeding (dsRNA*-TcGPCR*, dsRNA*-TcBAG*, and dsRNA*-TcGUK*) feeding were detected by slip-dip method. When the *TcGPCR*, *TcBAG* and *TcGUK* in the LC_50_ assays of scopoletin were knocked down by RNAi in *T. cinnabarinus*, mortality significantly decreased to 16.40% and significantly increased to 16.98, and 25.23% in mites fed with dsRNA*-TcGPCR*, dsRNA*-TcBAG*, and dsRNA*-TcGUK* compared with mites treated with DEPC–water, respectively (Figure 11). No significant mortality difference existed between DEPC–water and dsRNA-*GFP* (Figure 11). These results demonstrated that the RNAi of *TcGPCR* reduces the susceptibility of *T. cinnabarinus* to scopoletin and the RNAi of *TcBAG* and *TcGUK* enhences the susceptibility of *T. cinnabarinus* to scopoletin.

### Functional Assay

**FIGURE 12:**
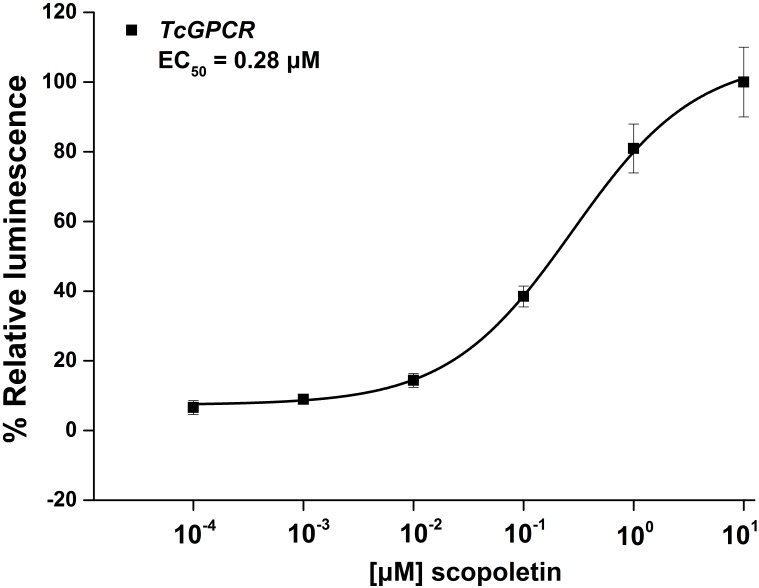
Relative activity of Chinese hamster ovary cells transfected with *T. cinnabarinus* G-protein-coupled neuropeptide receptor (GPCR) and activated with scopoletin. Concentration-response curves for luminescence induced by scopoletin. Results are means ± SD (*n* = 3).

To confirm that scopoletin induced an increase in intracellular free calcium [Ca^2+^]i levels by activating *TcGPCR*, we performed a cell-based assay with intracellular calcium mobilization in CHO cells. The results showed that a significant increase in intracellular calcium level in CHO cells expressing *TcGPCR* was induced by scopoletin in a concentration-dependent manner with a very low 50% effective concentration (EC_50_) value of 0.28 μM (Figure 12).

## Discussion

In this study, the effect of scopoletin on intracellular free calcium [Ca^2+^]i levels in insect Sf9 cells was investigated. We found that a significant increase in intracellular calcium level in insect Sf9 cells was induced by scopoletin in a concentration-dependent manner, indicating that the mode of action of scopoletin in insects was by inducing intracellular calcium overload. Meanwhile, the combination of Ca^2+^ and scopoletin can significantly improve its acaricidal activity (Hou et al., 2015), suggesting that the acaricidal mechanism of scopoletin involves calcium overload. As an important phenolic phytoalexin in plants, scopoletin presents numerous pharmacological activities, such as antitumor activity. Scopoletin also exerts its effect on inducing apoptosis of tumor cells by increasing intracellular calcium concentration (Manuele et al., 2006; Happi et al., 2012).

Thus, to search for candidate target genes of scopoletin inducing calcium overload in *T. cinnabarinus*, we applied transcriptomics on *T. cinnabarinus* treated with scopoletin or the solvent. A total of 52,496,305 and 52,286,859 reads were obtained from *T. cinnabarinus* treated with scopoletin or solvent at 24 and 48 h post-treatment, respectively. The obtained reads were mapped to the genome of *T. urticae* Koch, a sister species of *T. cinnabarinus* (Ueckermann et al., 2013). More than 80% of the reads were successfully mapped to the reference genome, thereby providing good mapping results for downstream analysis. We identified 70 and 102 differentially expressed genes upon scopoletin treatment at 24 and 48 h post-treatment, respectively. The number of upregulated genes was markedly higher than that of downregulated genes at 24 and 48 h post-treatment, thereby suggesting that more genes were activated by scopoletin at the two time points. However, the fold changes of certain differentially expressed genes, such as guanylate kinase, senescence-associated protein, and exostosin-1, were different between the two time points. The differential expression is closely related to a previous report that the poisoning symptoms of *T. cinnabarinus* change after scopoletin treatment over time (Liang et al., 2011). These symptoms indicated that different defense or lethal responses may be involved in *T. cinnabarinus* treated with scopoletin at different time points.

In this study, GO enrichment analysis of differentially expressed genes showed that “cellular process” was the dominant group at both time points. KEGG pathways showed that “protein processing in endoplasmic reticulum” represented the major biochemical pathway at 24 and 48 h post-treatment, whereas calcium signaling pathway, MAPK signaling pathway, and fat digestion and absorption were well represented at 48 h post-treatment. Furthermore, we manually selected candidate genes associated with mite detoxification and acaricidal mechanism, such as signal transduction genes (e.g., GUK, GPCR, and glycerol-3-phosphate dehydrogenase) (Sato et al., 2016), cell apoptosis genes (e.g., Bcl-2 protein, RAB5-interacting protein, HSP70, and prohibitin 2) (Hoffenberg et al., 2000; Kasashima et al., 2006), and energy metabolism genes (e.g., lipase, vitellogenin1, C4-dicarboxylate-binding protein, and AMP-dependent synthetase and ligase) (Dinh et al., 2002; Kawakami et al., 2009; Shaw et al., 2010; Tanaka et al., 2017), according to their biological functions. Among the selected candidate genes, genes associated with signal transduction and cell apoptosis were dominant. In addition, the differential expression of 2 transcription factor genes (e.g., transcription factor BCFI and SOX-2) was induced by scopoletin, indicating that the transcription factors were involved in the regulation of gene expression in the acaricidal mechanism of scopoletin.

Among the differentially expressed signal transduction genes identified in our study, GPCR was upregulated in *T. cinnabarinus* upon scopoletin treatment. Moreover, specific expression detection showed that scopoletin treatment upregulates the expression level of *TcGPCR*. GPCR composes one of the largest families of cell-surface proteins which involve in signal transmission and play crucial roles in diverse processes, such as development, metabolism, ecdysis, and reproduction in insects (Monaci et al., 1990; Van et al., 2010). GPCR can activate calcium channels present in the membrane of the endoplasmic reticulum, which induces the release of calcium into the cytoplasm (Caers et al., 2014). Thus, in the present study, to confirm that scopoletin induced an increase in intracellular free calcium [Ca^2+^]i levels by activating *TcGPCR*, we performed a cell-based assay with intracellular calcium mobilization in CHO cells. Indeed, the pharmacological data demonstrated that a significant increase in intracellular calcium level in CHO cells expressing *TcGPCR* was induced by scopoletin in a dose-dependent manner. Moreover, in this study, the susceptibility to scopoletin decreases when *TcGPCR* in the LC_50_ assays is suppressed via RNAi, indicating that the downregulation of GPCR reduces susceptibility to scopoletin. Taken all together, these results suggested that the calcium overload in the scopoletin-treated mites was mediated by the overexpression of GPCR.

In addition, the downregulation of GUK by scopoletin was observed in this study. Moreover, specific expression detection showed that scopoletin treatment downregulates the expression of *TcGUK*. GUK belongs to the superfamily of the membrane-associated guanylate kinase (MAGUK), which forms a complex with Ca^2+^ efflux pump of the plasma membrane Ca^2+^-ATPase (PMCA) to regulate calcium homeostasis (Aravindan et al., 2012). PMCA is responsible for the expulsion of Ca^2+^ from the cytosol of all eukaryotic cells (Zoccola et al., 2004). Aravindan et al. (2012) reported that GUK removes excess Ca^2+^ from cells by positively regulating the activity of PMCA. Elevated Ca^2+^ may result from increased influx or decreased efflux. Meanwhile, in this study, the susceptibility to scopoletin raises when *TcGUK* in the LC_50_ assays are suppressed via RNAi, indicating that the downregulation of GUK increases susceptibility to scopoletin. Thus, in our study, these results suggested that the downregulation of GUK may result in the closure of the efflux channel of calcium in the cell membrane, thereby inhibiting the outflow of intracellular Ca^2+^, which disrupts calcium homeostasis and promotes the overload of intracellular calcium. However, the regulation of intracellular calcium signaling is extremely complex. Therefore, the mechanism by which GUK downregulation mediates calcium overload in the scopoletin-treated mites needs further elucidation.

We identified several genes that were inhibited by scopoletin, including the apoptosis regulatory protein, Bcl-2 protein (BAG). Moreover, specific expression detection showed that scopoletin treatment downregulates the expression of *TcBAG*. Bcl-2 protein is a pro-survival protein that inhibits apoptosis induced by calcium signaling (Shibasaki et al., 1997). For example, the anti-apoptotic action of Bcl-2 reportedly involves enhancing the storage of calcium by upregulating the expression levels of calcium pump genes (Shibasaki et al., 1997; Zhu et al., 1999). Other reports indicate that Bcl-2 increases membrane permeability, thereby reducing the concentration of Ca^2+^ in the endoplasmic reticulum, resulting in a decrease in the amount of released Ca^2+^ during signal transduction and inhibiting apoptosis (Pinton et al., 2000; Schlossmann et al., 2000). Additionally, in this study, the susceptibility to scopoletin raises when *TcBAG* in the LC_50_ assays are suppressed via RNAi, indicating that the downregulation of Bcl-2 increases susceptibility to scopoletin. In this case, the Bcl-2 protein gene was inhibited by scopoletin, suggesting that the anti-apoptotic function induced by calcium signaling was disturbed in scopoletin-treated mites. Thus, the overloading of calcium induces cell apoptosis, and downregulation of Bcl-2 protein may promote apoptosis.

In our study, calcium signaling pathway-related genes (GPCR, BAG, and GUK) played crucial roles in the acaricidal mechanism of scopoletin against *T. cinnabarinus*. In consequence, the identification and characterization of calcium signaling pathway-related genes from mites will help in determining the involvement of GPCR, BAG, and GUK in the responses of mites to specific acaricides. Moreover, the present study will help us understand the biological functions of GPCR, BAG, and GUK. In this study, we cloned and characterized the full-length cDNA of GPCR, BAG, and GUK gene in *T. cinnabarinus* (designated as *TcGPCR*, *TcBAG*, and *TcGUK*, respectively). The structure analysis of *TcGPCR* demonstrates that this gene possesses seven transmembrane (7TM) helix domains that indicate the common structural framework of transmembrane signal transduction (Monaci et al., 1990). The structure analysis of *TcBAG* indicates that this gene possesses a BAG domain with anti-apoptotic activity and increases the anti-cell death function of Bcl-2 induced by calcium signaling (Shibasaki et al., 1997; Doong et al., 2002). Doong et al. (2002) reported that the gene was dependent on its interaction with heat shock protein 70 (HSP70) to exhibit anti-apoptotic activity. However, in this study, HSP70 was downregulated in BAG-suppressed mites, indicating that scopoletin promotes apoptosis. Moreover, *TcGUK* is predicted to possess a guanylate kinase-like domain (GK) whose function is to mediate the interaction of protein molecules, which is related to cell adhesion and orientation of mitotic spindle (Momand et al., 2000; Von et al., 2003; Yang et al., 2004). In addition, the expression levels of the calcium signaling pathway-related genes (*TcGPCR*, *TcBAG*, and *TcGUK*) were detected in all four tested developmental stages of *T. cinnabarinus*, indicating that the GPCR, BAG, and GUK genes are important during the whole life cycle of mites. However, the expression levels of the calcium signaling pathway-related genes (*TcGPCR*, *TcBAG*, and *TcGUK*) during the larval and nymphal stages were significantly higher than those in the other developmental stages of *T. cinnabarinus*, indicating that the three calcium signaling pathway-related genes coordinated and interacted during the regulation of mite development.

## Conclusion

In the present study, we found that the acaricidal mechanism of scopoletin involves calcium overload. Therefore, to reveal the molecular mechanism and search for candidate target genes of calcium overload induced by scopoletin in mites, we utilize RNA-seq to detect changes in transcription levels. We identified 70 and 102 differentially expressed genes upon scopoletin treatment at 24 and 48 h post-treatment, respectively. GO enrichment analysis of differentially expressed genes showed that “cellular process” was the dominant group at both time points. KEGG pathways showed that “protein processing in endoplasmic reticulum” represented the major biochemical pathway at 24 and 48 h post-treatment, whereas calcium signaling pathway, MAPK signaling pathway, and fat digestion and absorption were well represented at 48 h post-treatment. The target genes associated with the acaricidal mechanism of scopoletin included 3 signal transduction genes (GUK, GPCR, and glycerol-3-phosphate dehydrogenase), 4 cell apoptosis genes (Bcl-2 protein, RAB5-interacting protein, HSP70, and prohibitin 2), 4 energy metabolism genes (lipase, vitellogenin1, C4-dicarboxylate-binding protein, and AMP-dependent synthetase and ligase), and 2 transcription factor genes (transcription factor BCFI and SOX-2).

**FIGURE 13:**
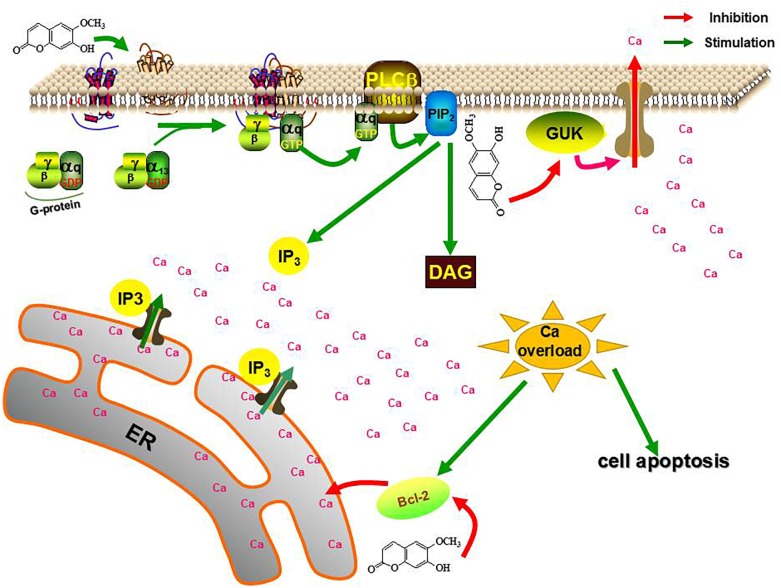
The role of *TcGPCR*, *TcBAG* and *TcGUK* on the acaricidal mechanism of scopoletin. PLCβ, phospholipase Cβ; PIP_2_, phosphatidylinositol bisphosphate; DAG, diacylglycerol; IP_3,_ inositol trisphosphate; ER, endoplasmic reticulum.

Mechanically, the calcium overload in the scopoletin-treated mites was mediated by calcium signaling pathway-related genes. Thus, the differential expression of three calcium signaling pathway-related genes, namely, GPCR, BAG, and GUK, may medicate calcium overload induced by scopoletin in RNA-seq. Specific expression detection shows that scopoletin treatment upregulates the expression levels of *TcGPCR* and downregulates the expression levels of *TcBAG* and *TcGUK*. Moreover, the RNAi of GPCR gene expression decreased the susceptibility of *T. cinnabarinus* to scopoletin, and the RNAi of BAG and GUK gene expressions enhanced the susceptibility of *T. cinnabarinus* to scopoletin. What is more, functional expression data strongly suggest that scopoletin induced a significant increase in intracellular free calcium [Ca^2+^]i levels by activating *TcGPCR* in CHO cells. Our results showed that the acaricidal mechanism of scopoletin against *T. cinnabarinus* was by disrupting intracellular Ca^2+^ homeostasis and calcium signaling pathway mediated by GPCR, BAG, and GUK (Figure 13). Our findings enhance the understanding of the acaricidal mechanism of scopoletin in *T. cinnabarinus* and clarify designing strategies to control pest mites.

## Author Contributions

HZ, Y-qZ, and WD conceived and designed the experiments and wrote and revised the manuscript. HZ, TL, X-jL, F-yG, and TG performed the experiments and analyzed the data.

## Conflict of Interest Statement

The authors declare that the research was conducted in the absence of any commercial or financial relationships that could be construed as a potential conflict of interest.

## Footnotes

1 https://www.ncbi.nlm.nih.gov/guide/genomes-maps/

2 http://bioinformatics.psb.ugent.be/orcae/overview/Teur

3 http://cn.expasy.org/tools/pi_tool.html

4 http://www.cbs.dtu.dk/services/SignalP/

5 http://www.cbs.dtu.dk/services/TMHMM/

6 http://www.cbs.dtu.dk/services/NetNGlyc/

7 http://frodo.wi.mit.edu/

8 http://www.originlab.com

9 http://www.ncbi.nlm.nih.gov/geo/

10 http://www.ncbi.nlm.nih.gov/
