# Supplementary material for: Acaricidal Mechanism of Scopoletin Against Tetranychus cinnabarinus
Source: Front Physiol. 2019 Mar 6;10:164. doi: 10.3389/fphys.2019.00164 (PMC6414448; doi:10.3389/fphys.2019.00164)
Supplement: Supplementary file 1 [file Presentation_1.ZIP › Supplemental materials.doc]

**Supplemental materials**

**Functional analysis reveals G-protein-coupled neuropeptide receptor-sensitive calcium release channel as a possible molecular target of scopoletin in the Carmine Spider Mite**

Hong Zhou^£^, Yong-qiang Zhang^£^, Ting Lai, Xue-jiao Liu, Fu-you Guo, Tao Guo & Wei Ding*

*Institute of Pesticide Science, College of Plant Protection, Southwest University, Chongqing 400715, P.R. China*

**Table S1 Primers used for cloning, qPCR, and RNAi**

| **Experiments** | **GenBank No. or Gene ID.** | **Genes** | **Primer names and sequences (5′-3′)** | **Product length (bp)** |
| --- | --- | --- | --- | --- |
| cloning | KY660538 | *TcGPCR* | F: ATGGAACCTTGGAATACAAATG | 1542 |
|  |  |  | R: TTACAGGGTTTCGGTCCCCGTT |  |
|  | KY660539 | *TcBAG* | F:ATGTCGCATTACAATAAAAGTT | 1857 |
|  |  |  | R:TTAATCAATCTTTTTCTGTTTC |  |
|  | KY660540 | *TcGUK* | F:ATGCTTTTATACCAAATTACTT | 336 |
|  |  |  | R:TTATTTAATCCAAATACTTGG |  |
| qPCR | FJ608659 | *RPS18* | F:ACGTGCTGGTGAACTTACCGAAGA | 99 |
|  |  |  | R:TGCCTATTCAAGAACCAAAGTGGG |  |
|  | tetur32g01740 | *TcBAG* | F:ACGCGATGCATGTTAAAGCT | 215 |
|  |  |  | R:TGCTGGACAGGTTCAGACTC |  |
|  | KY660540 | *TcGUK* | F:GGCTCCACCGACCAATATTG | 152 |
|  |  |  | R:TGTCAAAATGATGGCCAAAACG |  |
|  | KY660539 | Apoptosis regulator | F:ACGCGATGCATGTTAAAGCT | 215 |
|  |  |  | R:TGCTGGACAGGTTCAGACTC |  |
|  | tetur11g05740 | Lipase | F:TGCATCGTCCATTGAGCTTG | 219 |
|  |  |  | R:GCTCCATCGGCATGTTCAAT |  |
|  | tetur01g05810 | Exostosin-1 | F:ATCTTTACTTCGGAACTTGG | 61 |
|  |  |  | R:CCTCCTCTATCAGACTGTAT |  |
|  | tetur11g01680 | Heat shock protein 70 | F:CTGGCCGTTCAAAGTCATCA | 216 |
|  |  |  | R:AATAGCATAGACAGCCCGGG |  |
|  | tetur03g04050 | Thioredoxin-like protein 4A | F:AGATGGACGAAACACTCTATGGA | 173 |
|  |  |  | R:ACCAGTTCCCAGATCGATCA |  |
|  | tetur06g00140 | Senescence-associated protein | F:CTTTTGCCCTTTTGCTCTACG | 112 |
|  |  |  | R:ATTGTCAGGCGGGGAGTTT |  |
|  | KY660538 | G-protein coupled neuropeptide Receptor | F:ATGACTGGGCTGCCATCATA | 158 |
|  |  |  | R:AGTGATTGGTGTGGATGGGT |  |
|  | tetur201g00020 | Prohibitin 2 | F: ACAGCGTCAACAAGTTTCCC | 230 |
|  |  |  | R:CTTCTCCCTCAGCTTGGACA |  |
|  | tetur02g02600 | 26S protease regulatory subunit 6A | F:TGCAAATTCATTCGAGGCGA | 190 |
|  |  |  | R:GCCTGTACTTCCAAAATAGCGT |  |
|  | tetur08g07000 | AMP-dependent synthetase and ligase | F:GCGGCGATTTAGTGACCATT | 157 |
|  |  |  | R:ACACCAAATACTGCGGCATC |  |
|  | tetur17g02410 | ADP-ribosylation factor | F:AGATCGTCACAGAAGCAGCT | 166 |
|  |  |  | R:CTACTTGCAGCCCATTCGTC |  |
|  | tetur21g01620 | Similar to GA21569-PA | F:GGTAAATGTGGCCAACGTGT | 203 |
|  |  |  | R:GCGGGAGAGCGAATAACTTG |  |
|  | tetur04g07080 | Alpha-D-phosphohexomutase | F:GGAACTTCTGGACTACGAGC | 100 |
|  |  |  | R:CTTCAGCTGGGTTGTGGGAT |  |
|  | tetur651g00010 | Ribosomal protein S12 | F:ATGTCCATTCCGCTCTCCAA | 387 |
|  |  |  | R:AAACCGGCCCATTCTCCTAA |  |
| RNAi | KY660539 | *TcBAG* | F:taatacgactcactatagggAGGGTTTCGTTCCTCTTGTA | 551 |
|  |  |  | R:taatacgactcactatagggATTGGGTGGATTGACTGGTT |  |
|  | KY660538 | *TcGPCR* | F:taatacgactcactatagggATGGGCAAACCTCACAGTCG | 401 |
|  |  |  | R:taatacgactcactatagggTCCCCGTTGGTTTTATGG |  |
|  | KY660540 | *TcGUK* | F:taatacgactcactatagggATTGAGACATTGAAACGAGT | 230 |
|  |  |  | R:taatacgactcactatagggATACTTGGAATCCATTGAGG |  |
|  | ACY56286 | *GFP* | F:taatacgactcactatagggTCGGCGGCAATCCTGATCAA | 476 |
|  |  |  | R:taatacgactcactatagggTCACAGGGTAAAATTCAGCA |  |

**Table S2 Statistics of the reads generated from the RNA-seq and their mapping against *T. urticae*** genome

| **Sample^a^** | **Total Reads** | **Total Basepairs** | **Total Mapped Reads** | **Expressed Gene** | **Percentage of Mapping** |
| --- | --- | --- | --- | --- | --- |
| CK 24 h-1 | 13 083 313 | 1 838 765 016 | 12 714 468 | 14613 | 97.18% |
| CK 24 h-2 | 13 583 410 | 1 915 413 262 | 12 943 362 | 14777 | 95.29% |
| CK 48 h-1 | 12 748 755 | 1 810 155 299 | 11 990 145 | 14296 | 94.05% |
| CK 48 h-2 | 12 871 381 | 1 812 264 301 | 12 127 836 | 14559 | 94.22% |
| scopoletin 24 h-1 | 13 201 756 | 1 837 492 610 | 12 861 809 | 14764 | 97.42% |
| scopoletin 24 h-2 | 13 136 977 | 1 791 311 651 | 12 739 216 | 14578 | 96.97% |
| scopoletin 48 h-1 | 13 247 572 | 1 775 494 385 | 12 274 321 | 14449 | 92.65% |
| scopoletin 48 h-2 | 12 910 000 | 1 681 154 644 | 10 612 127 | 14482 | 82.20% |

^a^Two replicates for each treatment at each time point.

**Table S3 Screening significantly differentially expressed genes in *T. cinnabarinus* in response to scpoletin at 24 h posttreatment**

| **Gene ID** | **Description** | **RPKM** | | **Log_2_ fold change** | **Probability** |
| --- | --- | --- | --- | --- | --- |
|  |  | **CK** | **Scopoletin** |  |  |
| tetur13g03350 | Hypothetical protein | 5.8572 | 0.0100 | -9.1941 | 0.8792 |
| tetur16g01100 | Hypothetical protein | 13.7213 | 5.1344 | -1.4182 | 0.8236 |
| tetur04g03370 | Hypothetical protein | 1.3242 | 11.5957 | 3.1304 | 0.8858 |
| tetur15g00520 | hypothetical protein | 20.2529 | 45.5273 | 1.1686 | 0.8543 |
| tetur11g01880 | hypothetical protein | 2.8712 | 13.6383 | 2.2479 | 0.8718 |
| tetur02g06390 | Hypothetical protein | 8.0177 | 17.1256 | 1.0949 | 0.8006 |
| tetur21g00510 | RAB5-interacting protein | 16.3942 | 52.5307 | 1.6800 | 0.8969 |
| tetur03g04050 | Thioredoxin-like protein 4A | 15.6866 | 5.7149 | -1.4567 | 0.8366 |
| tetur19g01400 | Hypothetical protein | 11.7740 | 4.0100 | -1.5539 | 0.8226 |
| tetur32g02000 | Hypothetical protein | 83.7578 | 11.4684 | -2.8686 | 0.9408 |
| tetur03g06830 | Hypothetical protein | 13.9335 | 33.1899 | 1.2522 | 0.8536 |
| tetur27g01560 | Hypothetical protein | 4.6264 | 0.0100 | -8.8538 | 0.8505 |
| tetur08g03830 | EF-hand-like domain | 4.5785 | 12.0932 | 1.4012 | 0.8114 |
| tetur11g05740 | Lipase/lipooxygenase, PLAT/LH2 | 11.6255 | 1.4205 | -3.0328 | 0.8839 |
| tetur03g02140 | Hypothetical protein | 21.4616 | 9.9390 | -1.1106 | 0.8165 |
| tetur11g00380 | Hypothetical protein | 6.8681 | 15.7338 | 1.1959 | 0.8097 |
| tetur426g00010 | Hypothetical protein | 5.4782 | 15.0079 | 1.4539 | 0.8332 |
| tetur01g09640 | Hypothetical protein | 0.0100 | 13.0238 | 10.3469 | 0.9452 |
| tetur07g03400 | Hypothetical protein | 52.4144 | 25.9117 | -1.0164 | 0.8387 |
| tetur05g03470 | Hypothetical protein | 3.5005 | 0.0100 | -8.4514 | 0.8066 |
| tetur03g00200 | - | 9.2543 | 0.9526 | -3.2802 | 0.8716 |
| tetur07g05920 | Guanylate kinase | 46.8228 | 22.8164 | -1.0371 | 0.8389 |
| tetur03g00110 | - | 52.5321 | 11.0020 | -2.2554 | 0.9198 |
| tetur03g00210 | - | 39.9743 | 5.5027 | -2.8609 | 0.9293 |
| tetur01g05810 | exostosin-1 | 0.0100 | 18.0346 | 10.8166 | 0.9601 |
| tetur02g09970 | Hypothetical protein | 0.0100 | 4.3028 | 8.7491 | 0.8397 |
| tetur04g07140 | Hypothetical protein | 8.2859 | 20.8356 | 1.3303 | 0.8419 |
| tetur26g02170 | Hypothetical protein | 6.9344 | 0.8496 | -3.0289 | 0.8380 |
| tetur03g00120 | - | 54.7103 | 10.6184 | -2.3652 | 0.9238 |
| tetur06g06450 | Hypothetical protein | 2.8964 | 12.0305 | 2.0543 | 0.8545 |
| tetur35g01676 | hypothetical protein | 17.8328 | 7.0835 | -1.3320 | 0.8334 |
| tetur04g00270 | Hypothetical protein | 0.0100 | 13.6205 | 10.4116 | 0.9473 |
| tetur37g00540 | Hypothetical protein | 2.1772 | 13.1212 | 2.5914 | 0.8804 |
| tetur06g01130 | Hypothetical protein | 0.8234 | 6.3736 | 2.9525 | 0.8265 |
| tetur09g02040 | Hypothetical protein | 0.0100 | 3.8230 | 8.5786 | 0.8223 |
| tetur10g01920 | Hypothetical protein | 8.5336 | 2.4262 | -1.8145 | 0.8097 |
| tetur11g01680 | heat shock protein 70 | 128.5005 | 63.8484 | -1.0091 | 0.8528 |
| tetur95g00030 | Hypothetical protein | 0.0100 | 4.6924 | 8.8742 | 0.8522 |
| tetur15g00100 | Hypothetical protein | 0.5929 | 6.3991 | 3.4319 | 0.8374 |
| tetur10g02400 | Hypothetical protein | 22.1243 | 10.7913 | -1.0358 | 0.8086 |
| tetur06g00140 | senescence-associated protein | 242.9565 | 107.2315 | -1.1800 | 0.8778 |
| tetur01g01280 | Hypothetical protein | 6.9137 | 16.0478 | 1.2148 | 0.8130 |
| tetur34g00920 | Hypothetical protein | 0.2792 | 5.9615 | 4.4162 | 0.8489 |
| tetur09g01280 | Hypothetical protein | 1.0023 | 11.9813 | 3.5794 | 0.8947 |
| tetur01g07890 | - | 18.1118 | 7.0215 | -1.3671 | 0.8383 |
| tetur06g03690 | Hypothetical protein | 1.5150 | 7.4348 | 2.2949 | 0.8216 |
| tetur02g04640 | Hypothetical protein | 1.3796 | 6.7676 | 2.2944 | 0.8112 |
| tetur02g04740 | Hypothetical protein | 2.8148 | 10.7958 | 1.9394 | 0.8402 |
| tetur02g04010 | Hypothetical protein | 0.0100 | 5.7498 | 9.1674 | 0.8772 |
| tetur08g07940 | - | 47.5061 | 4.5305 | -3.3904 | 0.9410 |
| tetur14g00750 | Hypothetical protein | 6.9533 | 19.5867 | 1.4941 | 0.8523 |
| tetur02g13930 | Hypothetical protein | 2.1557 | 7.6991 | 1.8365 | 0.8006 |
| tetur03g03140 | Hypothetical protein | 0.0100 | 8.3665 | 9.7085 | 0.9150 |
| tetur01g08530 | Hypothetical protein | 1.3264 | 7.1707 | 2.4345 | 0.8232 |
| tetur02g05380 | scaffold protein | 0.0100 | 5.9000 | 9.2046 | 0.8800 |
| tetur32g01740 | Apoptosis regulator, Bcl-2 protein, BAG | 8.4983 | 1.6847 | -2.3347 | 0.8364 |
| tetur15g00490 | Hypothetical protein | 0.0100 | 10.5594 | 10.0443 | 0.9327 |
| tetur02g02050 | C4-dicarboxylate-binding protein | 1.7990 | 7.9400 | 2.1419 | 0.8212 |
| tetur04g01410 | Hypothetical protein | 0.0100 | 5.1788 | 9.0165 | 0.8650 |
| tetur05g07850 | hypothetical protein | 11.4238 | 4.2319 | -1.4327 | 0.8091 |
| tetur19g01170 | Hypothetical protein | 1.9941 | 14.1583 | 2.8278 | 0.8911 |
| tetur18g02520 | Hypothetical protein | 4.1400 | 20.0673 | 2.2772 | 0.8929 |
| tetur03g01870 | Hypothetical protein | 16.9521 | 35.0082 | 1.0462 | 0.8309 |
| tetur04g09380 | Hypothetical protein | 7.1084 | 0.5939 | -3.5812 | 0.8502 |
| tetur18g01220 | Hypothetical protein | 1.2747 | 6.3463 | 2.3157 | 0.8052 |
| tetur03g02030 | Hypothetical protein | 0.2103 | 4.7566 | 4.4994 | 0.8226 |
| tetur17g03730 | Hypothetical protein | 5.1803 | 0.2092 | -4.6302 | 0.8363 |
| tetur02g06010 | Hypothetical protein | 5.7201 | 0.4923 | -3.5383 | 0.8266 |
| tetur33g01110 | Hypothetical protein | 9.1095 | 2.7376 | -1.7345 | 0.8111 |
| tetur18g00810 | Hypothetical protein | 52.8305 | 137.3630 | 1.3786 | 0.8927 |

**Table S4 Screening significantly differentially expressed genes in *T. cinnabarinus* in response to scpoletin at 48 h posttreatment**

| **Gene ID** | **Description** | **RPKM** | | **Log_2_ fold change** | **Probability** |
| --- | --- | --- | --- | --- | --- |
|  |  | **CK** | **Scopoletin** |  |  |
| tetur11g05420 | Hypothetical protein | 37.2506 | 16.6984 | -1.1576 | 0.8234 |
| tetur08g02600 | Hypothetical protein | 0.8584 | 9.5855 | 3.4811 | 0.8558 |
| tetur08g07000 | AMP-dependent synthetase and ligase | 9.9772 | 0.8585 | -3.5387 | 0.8613 |
| tetur03g04130 | Hypothetical protein | 0.0100 | 4.6993 | 8.8763 | 0.8358 |
| tetur19g01390 | Hypothetical protein | 0.0100 | 5.0684 | 8.9854 | 0.8457 |
| tetur02g00650 | Hypothetical protein | 0.0100 | 58.7325 | 12.5199 | 0.9854 |
| tetur23g01700 | - | 68.6523 | 24.9983 | -1.4575 | 0.8709 |
| tetur17g02780 | Hypothetical protein | 0.0100 | 5.3970 | 9.0760 | 0.8544 |
| tetur15g01650 | Hypothetical protein | 7.1639 | 17.4493 | 1.2844 | 0.8015 |
| tetur32g02000 | Hypothetical protein | 106.5050 | 26.0565 | -2.0312 | 0.9068 |
| tetur201g00020 | prohibitin 2 | 8.6853 | 0.5558 | -3.9660 | 0.8580 |
| tetur36g00010 | - | 6.1020 | 23.0290 | 1.9161 | 0.8637 |
| tetur66g00140 | hypothetical protein | 13.3542 | 89.8907 | 2.7509 | 0.9255 |
| tetur04g02050 | hypothetical protein IscW_ISCW020080 | 1.2624 | 10.1899 | 3.0129 | 0.8512 |
| tetur01g09640 | Hypothetical protein | 0.0100 | 20.8705 | 11.0272 | 0.9599 |
| tetur08g00020 | Hypothetical protein | 4.9390 | 13.9474 | 1.4977 | 0.8057 |
| tetur02g00040 | - | 13.3497 | 4.2351 | -1.6563 | 0.8147 |
| tetur02g03830 | NP-R8:G-protein coupled neuropeptide receptor | 3.0684 | 18.2442 | 2.5719 | 0.8782 |
| tetur13g00390 | Hypothetical protein | 30.3405 | 12.1493 | -1.3204 | 0.8359 |
| tetur01g05420 | BmGATA-beta:Transcription factor BCFI | 3.9215 | 15.9409 | 2.0233 | 0.8495 |
| tetur418g00020 | Hypothetical protein | 6.7851 | 43.7166 | 2.6877 | 0.9127 |
| tetur07g05920 | Guanylate kinase | 71.2255 | 0.0100 | -12.7982 | 0.9882 |
| tetur29g01470 | unnamed protein product | 0.0100 | 14.4343 | 10.4953 | 0.9418 |
| tetur03g00110 | - | 48.4784 | 8.3613 | -2.5355 | 0.9112 |
| tetur18g03360 | hypothetical protein | 2.0944 | 9.5410 | 2.1876 | 0.8164 |
| tetur22g02960 | Hypothetical protein | 12.4301 | 3.5863 | -1.7933 | 0.8182 |
| tetur38g00600 | Hypothetical protein | 27.5559 | 12.5567 | -1.1339 | 0.8055 |
| tetur05g06160 | Hypothetical protein | 25.4544 | 0.0775 | -8.3593 | 0.9642 |
| tetur03g00210 | - | 38.6031 | 12.0379 | -1.6811 | 0.8717 |
| tetur14g01500 | Hypothetical protein | 10.4840 | 1.0372 | -3.3375 | 0.8607 |
| tetur01g05810 | exostosin-1 | 0.0100 | 22.5082 | 11.1362 | 0.9629 |
| tetur09g07490 | - | 43.8619 | 21.7151 | -1.0143 | 0.8056 |
| tetur17g00700 | Hypothetical protein | 22.4352 | 7.2058 | -1.6385 | 0.8469 |
| tetur107g00010 | Hypothetical protein | 0.1076 | 8.7899 | 6.3524 | 0.8903 |
| tetur11g01430 | PREDICTED: similar to Negative elongation factor E CG5994-PA | 45.0860 | 94.5915 | 1.0690 | 0.8331 |
| tetur02g10020 | Hypothetical protein | 29.5535 | 13.6855 | -1.1107 | 0.8053 |
| tetur26g02170 | Hypothetical protein | 0.7001 | 6.7596 | 3.2712 | 0.8168 |
| tetur12g01770 | Hypothetical protein | 0.0100 | 37.9382 | 11.8894 | 0.9777 |
| tetur03g00120 | - | 48.1848 | 13.5264 | -1.8328 | 0.8856 |
| tetur02g02600 | 26S protease regulatory subunit 6A | 13.2758 | 1.6563 | -3.0028 | 0.8714 |
| tetur04g00270 | Hypothetical protein | 0.0100 | 36.3096 | 11.8261 | 0.9769 |
| tetur16g03140 | Hypothetical protein | 3.1119 | 11.6559 | 1.9052 | 0.8191 |
| tetur02g12070 | transcription factor SOX-2 | 17.6862 | 6.0849 | -1.5393 | 0.8265 |
| tetur38g00520 | Hypothetical protein | 0.0100 | 37.5307 | 11.8739 | 0.9776 |
| tetur17g03950 | Hypothetical protein | 20.8790 | 8.8494 | -1.2384 | 0.8075 |
| tetur15g01820 | DM:DM DNA binding domain | 29.7538 | 12.7198 | -1.2260 | 0.8241 |
| tetur01g05010 | Hypothetical protein | 0.0100 | 3.7953 | 8.5681 | 0.8053 |
| tetur23g01300 | glycerol-3-phosphate dehydrogenase | 9.9892 | 2.5329 | -1.9796 | 0.8101 |
| tetur07g01210 | Hypothetical protein | 0.0100 | 97.4605 | 13.2506 | 0.9913 |
| tetur17g00980 | Hypothetical protein | 27.0198 | 9.6772 | -1.4814 | 0.8445 |
| tetur08g05180 | Hypothetical protein | 0.0100 | 12.3863 | 10.2745 | 0.9328 |
| tetur03g08460 | Hypothetical protein | 0.0100 | 213.6160 | 14.3827 | 0.9961 |
| tetur12g01310 | Hypothetical protein | 4.0891 | 13.7076 | 1.7451 | 0.8233 |
| tetur06g01630 | Hypothetical protein | 1.5154 | 13.1759 | 3.1202 | 0.8735 |
| tetur04g07240 | - | 15.6148 | 0.0100 | -10.6087 | 0.9459 |
| tetur01g14420 | Hypothetical protein | 5.9955 | 16.2035 | 1.4344 | 0.8110 |
| tetur10g01420 | Hypothetical protein | 0.0100 | 4.0712 | 8.6693 | 0.8163 |
| tetur95g00030 | Hypothetical protein | 0.0100 | 12.2842 | 10.2626 | 0.9322 |
| tetur01g01530 | Hypothetical protein | 2.6386 | 11.4540 | 2.1180 | 0.8297 |
| tetur16g00460 | Hypothetical protein | 6.6972 | 16.7196 | 1.3199 | 0.8027 |
| tetur01g06630 | Hypothetical protein | 0.5865 | 7.6291 | 3.7012 | 0.8392 |
| tetur06g00140 | senescence-associated protein | 266.4575 | 38.5752 | -2.7882 | 0.9342 |
| tetur651g00010 | ribosomal protein S12 | 21.0008 | 4.4955 | -2.2239 | 0.8736 |
| tetur03g00260 | Hypothetical protein | 0.0100 | 8.7029 | 9.7654 | 0.9051 |
| tetur10g05120 | Hypothetical protein | 9.6253 | 2.5646 | -1.9081 | 0.8012 |
| tetur29g00900 | Hypothetical protein | 0.0100 | 10.3116 | 10.0101 | 0.9201 |
| tetur39g00730 | vitellogenin1 | 17.4034 | 4.1871 | -2.0553 | 0.8558 |
| tetur08g06300 | ADP-ribosylation factor-like protein 6 | 6.2265 | 30.8088 | 2.3068 | 0.8927 |
| tetur20g00520 | Hypothetical protein | 0.0100 | 20.3752 | 10.9926 | 0.9591 |
| tetur118g00010 | - | 0.0737 | 8.1843 | 6.7960 | 0.8873 |
| tetur03g08830 | - | 125.4170 | 59.5280 | -1.0751 | 0.8389 |
| tetur03g02110 | Hypothetical protein | 0.0100 | 6.3822 | 9.3179 | 0.8741 |
| tetur54g00060 | Hypothetical protein | 3.6857 | 11.6457 | 1.6598 | 0.8030 |
| tetur04g07080 | Alpha-D-phosphohexomutase, alpha/beta/alpha I/II/III | 0.0100 | 109.2025 | 13.4147 | 0.9922 |
| tetur02g04010 | Hypothetical protein | 0.0100 | 5.0161 | 8.9704 | 0.8441 |
| tetur12g04490 | DEAD-box ATP dependent DNA helicase | 2.5244 | 11.3534 | 2.1691 | 0.8311 |
| tetur01g01290 | Hypothetical protein | 28.0936 | 10.5469 | -1.4134 | 0.8402 |
| tetur19g01410 | Hypothetical protein | 0.0100 | 8.8612 | 9.7914 | 0.9068 |
| tetur08g07940 | - | 45.4389 | 13.0697 | -1.7977 | 0.8830 |
| tetur03g08470 | Hypothetical protein | 0.0100 | 213.6160 | 14.3827 | 0.9961 |
| tetur04g04510 | Hypothetical protein | 5.7124 | 0.0100 | -9.1580 | 0.8612 |
| tetur08g03190 | Hypothetical protein | 0.3860 | 5.4676 | 3.8243 | 0.8039 |
| tetur01g10050 | Hypothetical protein | 2.6880 | 14.1737 | 2.3986 | 0.8583 |
| tetur05g04350 | dolichyl-diphosphooligosaccharide--protein glycosyltransferase subunit 4 | 32.6193 | 4.6704 | -2.8041 | 0.9087 |
| tetur03g03140 | Hypothetical protein | 0.0100 | 3.8475 | 8.5878 | 0.8080 |
| tetur02g05380 | scaffold protein | 9.1194 | 0.8489 | -3.4253 | 0.8505 |
| tetur23g00420 | Hypothetical protein | 0.0100 | 27.4611 | 11.4232 | 0.9701 |
| tetur02g08470 | conserved hypothetical protein | 0.0483 | 5.6733 | 6.8774 | 0.8492 |
| tetur15g00490 | Hypothetical protein | 0.0100 | 14.7947 | 10.5309 | 0.9431 |
| tetur02g02050 | C4-dicarboxylate-binding protein | 6.6234 | 0.0100 | -9.3714 | 0.8777 |
| tetur01g01120 | Hypothetical protein | 0.0100 | 3.6875 | 8.5265 | 0.8009 |
| tetur13g00180 | Hypothetical protein | 20.3639 | 8.4613 | -1.2671 | 0.8099 |
| tetur17g02410 | ADP-ribosylation factor GTPase-activating protein 1 | 0.0914 | 4.5619 | 5.6411 | 0.8109 |
| tetur21g00220 | Hypothetical protein | 0.0100 | 5.3246 | 9.0565 | 0.8526 |
| tetur19g02350 | Hypothetical protein | 14.5577 | 5.4543 | -1.4163 | 0.8015 |
| tetur13g00050 | - | 14.7381 | 0.1795 | -6.3596 | 0.9271 |
| tetur04g07530 | Hypothetical protein | 8.4889 | 1.6616 | -2.3530 | 0.8124 |
| tetur31g00280 | conserved hypothetical protein | 15.5082 | 5.2595 | -1.5600 | 0.8193 |
| tetur21g01620 | PREDICTED: similar to GA21569-PA | 0.1296 | 6.2176 | 5.5846 | 0.8493 |
| tetur01g03980 | Hypothetical protein | 0.0100 | 6.0841 | 9.2489 | 0.8689 |
| tetur03g08450 | Hypothetical protein | 0.0100 | 213.6160 | 14.3827 | 0.9961 |
| tetur01g16564 | hypothetical protein | 67.7776 | 150.6040 | 1.1519 | 0.8500 |

***Table S5 Complete sequence information of the GPCR, GUK and BAG genes of*** T. cinnabarinus

| **Gene** | **Accession numbers** | **Coding sequence (bp)** | **Deduced amino acid** | **Calculated molecular (kDa)** | **Isoelectric point** |
| --- | --- | --- | --- | --- | --- |
|  |  |  |  |  |  |
|  |  |  |  |  |  |
| *TcGPCR* | KY660538 | 1542 | 513 | 131.38 | 4.77 |
| *TcGUK* | KY660540 | 336 | 111 | 27.9 | 5.17 |
| *TcBAG* | KY660539 | 1857 | 618 | 154.98 | 4.74 |

**Table S6 Sequences and relevant information for phylogenetic analysis of *TcGPCR, TcBAG and TcGUK***

| Genes | GenBank No. or Gene ID. | Species |
| --- | --- | --- |
| *SsGPCR* | *Sarcoptes scabiei* | KPM10590.1 |
| *DmGPCR* | *Daphnia magna* | JAN54480.1 |
| *PcGPCR* | *Pediculus humanus corporis* | XP_002432135.1 |
| *PdGPCR* | *Platynereis dumerilii* | AKQ63023.1 |
| *LaGPCR* | *Lingula anatina* | XP_013413429.1 |
| *AsGPCR* | *Amblyomma sculptum* | JAU01058.1 |
| *NlGPCR* | *Nilaparvata lugens* | BAO01092.1 |
| *PtGPCR* | *Parasteatoda tepidariorum* | [XP_015910982.1](https://www.ncbi.nlm.nih.gov/protein/1009550781?report=genbank&log$=prottop&blast_rank=56&RID=AX7HXCEE014) |
| *TpGPCR* | *Trichinella pseudospiralis* | [KRY72198.1](https://www.ncbi.nlm.nih.gov/protein/954420686?report=genbank&log$=prottop&blast_rank=79&RID=AX7HXCEE014) |
| *TnGPCR* | *Trichinella nelsoni* | [KRX20640.1](https://www.ncbi.nlm.nih.gov/protein/954211430?report=genbank&log$=prottop&blast_rank=99&RID=AX7HXCEE014) |
| *TsGPCR* | *Trichinella spiralis* | [KRY35674.1](https://www.ncbi.nlm.nih.gov/protein/954369403?report=genbank&log$=prottop&blast_rank=100&RID=AX7HXCEE014) |
| *TcGPCR* | *Tetranychus cinnabarinus* | KY660538 |
| *TuGPCR* | *Tetranychus urticae* | tetur02g03830 |
| *ApBAG* | *Agrilus planipennis* | XP_018325480.1 |
| *LpBAG* | *Limulus polyphemus* | [XP_013784769.1](https://www.ncbi.nlm.nih.gov/protein/926637572?report=genbank&log$=prottop&blast_rank=8&RID=AX8XB4V8014) |
| *ObBAG* | *Operophtera brumata* | KOB74632.1 |
| *BcBAG* | *Bactrocera cucurbitae* | [XP_011190500.1](https://www.ncbi.nlm.nih.gov/protein/751440599?report=genbank&log$=prottop&blast_rank=12&RID=AX8XB4V8014) |
| *AgBAG* | *Anoplophora glabripennis* | [XP_018566388.1](https://www.ncbi.nlm.nih.gov/protein/1080049918?report=genbank&log$=prottop&blast_rank=16&RID=AX8XB4V8014) |
| *MqBAG* | *Melipona quadrifasciata* | [KOX81288.1](https://www.ncbi.nlm.nih.gov/protein/925684838?report=genbank&log$=prottop&blast_rank=17&RID=AX8XB4V8014) |
| *OcBAG* | *Orchesella cincta* | [ODN02430.1](https://www.ncbi.nlm.nih.gov/protein/1061485290?report=genbank&log$=prottop&blast_rank=19&RID=AX8XB4V8014) |
| *PtBAG* | *Parasteatoda tepidariorum* | [XP_015903931.1](https://www.ncbi.nlm.nih.gov/protein/1009533887?report=genbank&log$=prottop&blast_rank=21&RID=AX8XB4V8014) |
| *BdBAG* | *Bactrocera dorsalis* | [XP_011200616.1](https://www.ncbi.nlm.nih.gov/protein/751782792?report=genbank&log$=prottop&blast_rank=22&RID=AX8XB4V8014) |
| *CcBAG* | *Ceratitis capitata* | [XP_012162305.1](https://www.ncbi.nlm.nih.gov/protein/807044439?report=genbank&log$=prottop&blast_rank=25&RID=AX8XB4V8014) |
| *RzBAG* | *Rhagoletis zephyria* | [XP_017479638.1](https://www.ncbi.nlm.nih.gov/protein/1048049171?report=genbank&log$=prottop&blast_rank=34&RID=AX8XB4V8014) |
| *TcBAG* | *Tetranychus cinnabarinus* | KY660539 |
| *TuBAG* | *Tetranychus urticae* | tetur32g01740 |
| *SsGUK* | *Sarcoptes scabiei* | [KPM08071.1](https://www.ncbi.nlm.nih.gov/protein/934158859?report=genbank&log$=prottop&blast_rank=4&RID=AXAAKNVK015) |
| *SmGUK* | *Stegodyphus mimosarum* | KFM66744.1 |
| *AtGUK* | *Aethina tumida* | [XP_019869597.1](https://www.ncbi.nlm.nih.gov/protein/1133427468?report=genbank&log$=prottop&blast_rank=6&RID=AXAAKNVK015) |
| *DpGUK* | *Dendroctonus ponderosae* | XP_019760820.1 |
| *PmGUK* | *Papilio machaon* | [KPJ15670.1](https://www.ncbi.nlm.nih.gov/protein/930675411?report=genbank&log$=prottop&blast_rank=10&RID=AXAAKNVK015) |
| *PxGUK* | *Papilio xuthus* | XP_013162147.1 |
| *LpGUK* | *Limulus polyphemus* | XP_013778768.1 |
| *ApGUK* | *Agrilus planipennis* | XP_018322329.1 |
| *PpGUK* | *Papilio polytes* | XP_013136134.1 |
| *AgGUK* | *Anoplophora glabripennis* | [XP_018564866.1](https://www.ncbi.nlm.nih.gov/protein/1080047151?report=genbank&log$=prottop&blast_rank=20&RID=AXAAKNVK015) |
| *CfGUK* | *Copidosoma floridanum* | [XP_014217886.1](https://www.ncbi.nlm.nih.gov/protein/936607782?report=genbank&log$=prottop&blast_rank=26&RID=AXAAKNVK015) |
| *TuGUK* | *Tetranychus urticae* | tetur07g05920 |
| *TcGUK* | *Tetranychus cinnabarinus* | KY660540 |

**Figure S1 Chemical structure of scopoletin.**

**Figure S2 Core structure of coumarins.**

**Figure S2 Sequencing saturation analysis of the number of detected genes from the RNA-seq in *T. cinnabarinus* after scopoletin or solvent treatment.**
